# 7-nm Mn_0.5_ Zn_0.5_Fe_2_O_4_ superparamagnetic iron oxide nanoparticle (SPION): a high-performance theranostic for MRI and hyperthermia applications

**DOI:** 10.7150/thno.103503

**Published:** 2025-02-10

**Authors:** Joo Young Lee, Yi Rang Na, Chul Min Na, Pyung Won Im, Hyung Woo Park, Min Kyu Kim, Yona Kim, Ji Hyeon You, Dong Su Kang, Hyo Eun Moon, Hye Ran Park, Min Gyu Kim, Pilhan Kim, Sung Hye Park, Hye Won Youn, Young Don Son, Yasushi Takemura, Chang Won Song, Daishun Ling, Yuanzhe Piao, Sun Ha Paek

**Affiliations:** 1 Department of Neurosurgery, Cancer Research Institute, Seoul National University College of Medicine. Seoul 03080, Republic of Korea.; 2 Translational Immunology Lab, Department of Transdisciplinary Medicine, Seoul National University Hospital, Seoul 03080, Republic of Korea.; 3 Immunology Core Facility, Department of Translational Research Center, Biomedical Research Institute, Seoul National University Hospital, Republic of; Korea.; 4 Department of Biomedical Sciences, Seoul National University College of Medicine, Seoul 03080, Republic of Korea.; 5 Department of Neurosurgery, Soonchunhyang University Seoul Hospital, Seoul 04401, Republic of Korea.; 6 Pohang Accelerator Laboratory (PAL) Pohang 37673, Republic of Korea.; 7 Graduate School of Medical Science and Engineering, Korea Advanced Institute of Science and Technology (KAIST), Daejeon, 34141, Republic of Korea.; 8 Korea Institute for Health Science and Technology (KIHST), Korea Advanced Institute of Science and Technology (KAIST), Daejeon, 34141, Republic of Korea.; 9 Department of Pathology, Neuroscience Research Institute, Seoul National University College of Medicine, Seoul 03080, Republic of Korea.; 10 Department of Nuclear Medicine, Seoul National University Hospital, Republic of Korea. Laboratory of Molecular Imaging and Therapy, Cancer Research Institute, Seoul National University College of Medicine, Republic of Korea.; 11 Department of Biomedical Engineering, Gachon University, Seongnam, Republic of Korea; 12 Department of Electrical and Computer Engineering, Yokohama National University Yokohama 240-8501, Japan.; 13 Department of Radiation Oncology, University of Minnesota Medical School, Minneapolis, Minnesota, USA.; 14 Frontiers Science Center for Transformative Molecules, School of Chemistry and Chemical Engineering, School of Biomedical Engineering, National Center for Translational Medicine, State Key Laboratory of Oncogenes and Related Genes, Shanghai Jiao Tong University, 200240, Shanghai, China; World Laureates Association (WLA) Laboratories, 201203, Shanghai, China.; 15 Department of Molecular Medicine and Biopharmaceutical Sciences, Graduate School of Seoul National University, Seoul 08826, Republic of Korea. Advanced Institutes of Convergence Technology, Suwon-si, Gyeonggi-do, Republic of Korea.; 16 Hypoxia/Ischemia Disease Institute, Seoul National University College of Medicine, Seoul 03080, Republic of Korea. Advanced Institute of Convergence Technology, Seoul National University, Suwon16229, Republic of Korea.

**Keywords:** alternating magnetic fields (AMFs), glioblastoma, magnetic hyperthermia, Mn_0.5_Zn_0.5_Fe_2_O_4_, superparamagnetic iron oxide nanoparticle (SPION)

## Abstract

Superparamagnetic iron oxide nanoparticles (SPIONs) are promising contrast agents for imaging-guided cancer therapies. However, challenges such as the requirement for a high alternating magnetic field (AMF), dosage limitations, and suboptimal imaging contrast have hindered their practical applications.

**Methods:** First, the optimal doping ratio of Mn and Zn in Mn_x_Zn_1-x_Fe_2_O_4_ nanoparticles synthesized using a modified high-temperature thermal decomposition method (mHTTD) was determined. Then, the magnetic and physical properties of the optimal 7-nm Mn_0.5_Zn_0.5_Fe_2_O_4_ SPIONs were systematically and comprehensively characterized via hysteresis measurements, dynamic light scattering (DLS), transmission electron microscopy (TEM), X-ray diffraction (XRD), X-ray absorption fine structure (XAFS) spectroscopy, and X-ray absorption near edge structure (XANES) spectroscopy. Next, the stability, biosafety, biocompatibility, and theranostic performance of 7-nm Mn_0.5_Zn_0.5_Fe_2_O_4_ SPIONs in magnetic hyperthermia therapy (MHT) were evaluated by *in vivo* and *in vitro* studies involving mouse models, magnetic resonance imaging (MRI), and bioassays. The results were then compared with those for conventional SPIONs.

**Results:** Under an AMF of 140 Oe at 100 kHz, 7-nm Mn_0.5_Zn_0.5_Fe_2_O_4_ SPIONs demonstrated significantly higher heat production than conventional SPIONs. Following surface modification with methoxy-PEG-silane, PEGylated 7-nm Mn_0.5_Zn_0.5_Fe_2_O_4_ SPIONs showed excellent monodispersity and magnetic properties, with an exceptionally high T2 relaxivity (r2).

**Conclusions:** The high *in vitro* and *in vivo* theranostic performance of PEGylated 7-nm Mn_0.5_Zn_0.5_Fe_2_O_4_ SPIONs as efficient and stable contrast agents for treating glioblastoma, encompassing strengthened magnetic hyperthermia, activated anti-tumor immunity, and remarkable T2 contrast enhancement, underscores the potential of precisely designed ferrites to concurrently enhance the T2 contrast and magnetocaloric properties for optimal theranostic outcomes. Our study provides a compelling rationale for the development of tailored magnetic nanoprobes for improved glioblastoma theranostics.

## Introduction

Nanomedicine has advanced significantly in recent years, enabling the use of nanoparticles (NPs) in cancer therapy with enhanced stability and improved safety across various tumor types 1. Among these innovations, magnetic hyperthermia therapy (MHT) has emerged as a promising alternative for treating tumors that are resistant to conventional therapies 2. MHT leverages the unique properties of magnetic NPs to provide non-invasive, highly penetrating, and remotely activated treatment options, making it particularly suitable for treating difficult-to-treat cancers.

Magnetic resonance imaging (MRI) has been widely adopted as a diagnostic and therapeutic monitoring tool because of its high-resolution and real-time capabilities. The integration of MHT with MRI has led to the development of theranostic NPs that enable simultaneous tumor visualization and localized hyperthermia, offering advantages for both treatment and monitoring 3, 4. This approach has shown significant promise, particularly for aggressive malignancies, such as glioblastoma, which is one of the most malignant and therapy-resistant brain tumors. However, MHT efficacy depends heavily on the power of the alternating magnetic fields (AMF) and/or the dosage of injected magnetic materials. Consequently, these requirements cause biomedical safety thresholds to be exceeded, raising concerns regarding potential collateral tissue damage.

Several types of NPs, including Gd-based silica and Au NPs, have been explored for their potential to seamlessly combine the MHT and MRI into a unified theranostic platform 5, 6. Superparamagnetic iron oxide NPs (SPIONs) have garnered significant attention in imaging-guided therapy because of their unique magnetic properties, relatively low toxicity, and cost-effectiveness 7-14. While early studies focused on adjusting the size of magnetic iron oxide (Fe_3_O_4_) NPs to enhance their magnetization (Ms) and magnetocaloric effects, this approach was constrained by the predominance of superparamagnetism in small SPIONs (< 20 nm) 2, 11-17. Recently, the incorporation of exogenous ions into the SPION matrix has been reported to yield spinel-structured ferrites with improved magnetic properties 18, 19. Typically, metal ions such as Zn^2+^, Mn^2+^, Ni^2+^ and Co^2+^ have been utilized to design ferrite NPs, allowing the modulation of their magnetic properties 20. In particular, Zn/Mn co-doped ferrite NPs can achieve high Ms values despite their small size, which enhances their magnetic responsiveness and leads to superior MHT performance 19, 21. Despite these advancements, only a few studies have optimized both the T2 contrast ability and the magnetocaloric effect of ferrite NPs through precise atomic doping to enhance the therapeutic responses in tumors.

In this study, we investigated different ratios of Zn/Mn co-doped ferrite NPs and synthesized 7-nm Mn_0.5_Zn_0.5_Fe_2_O_4_ SPION (MnZn-SPION-7) using a modified high-temperature thermal decomposition method (mHTTD) that incorporates manganese and zinc into the Fe_3_O_4_ matrix. When subjected to alternating current (AC) magnetic fields, the MnZn-SPION-7 matrix exhibited oscillating structural changes facilitated by the O atoms surrounding Fe ions within the spinel structure. This dynamic response effectively promoted hyperthermia reaction. Upon modification with methoxy-PEG-silane, the resulting PEGylated MnZn-SPION-7s (PEG-MnZn-SPION-7) demonstrated excellent monodispersity and retained its magnetic properties in the aqueous phase. Particularly, these nanoprobes exhibited remarkably high T2 relaxivity (r2). Subsequently, we evaluated their potential for enhanced MHT and initiation of anti-tumor immunity in glioblastoma, both *in vitro* and *in vivo*. Our results demonstrate the diagnostic and therapeutic capabilities of the Mn_0.5_Zn_0.5_Fe_2_O_4_ NPs and their potential theranostic applications.

### Optimal SPION Mn/Zn ratio is Mn_0.5_Zn_0.5_Fe_2_O_4_

**Figure 1:**
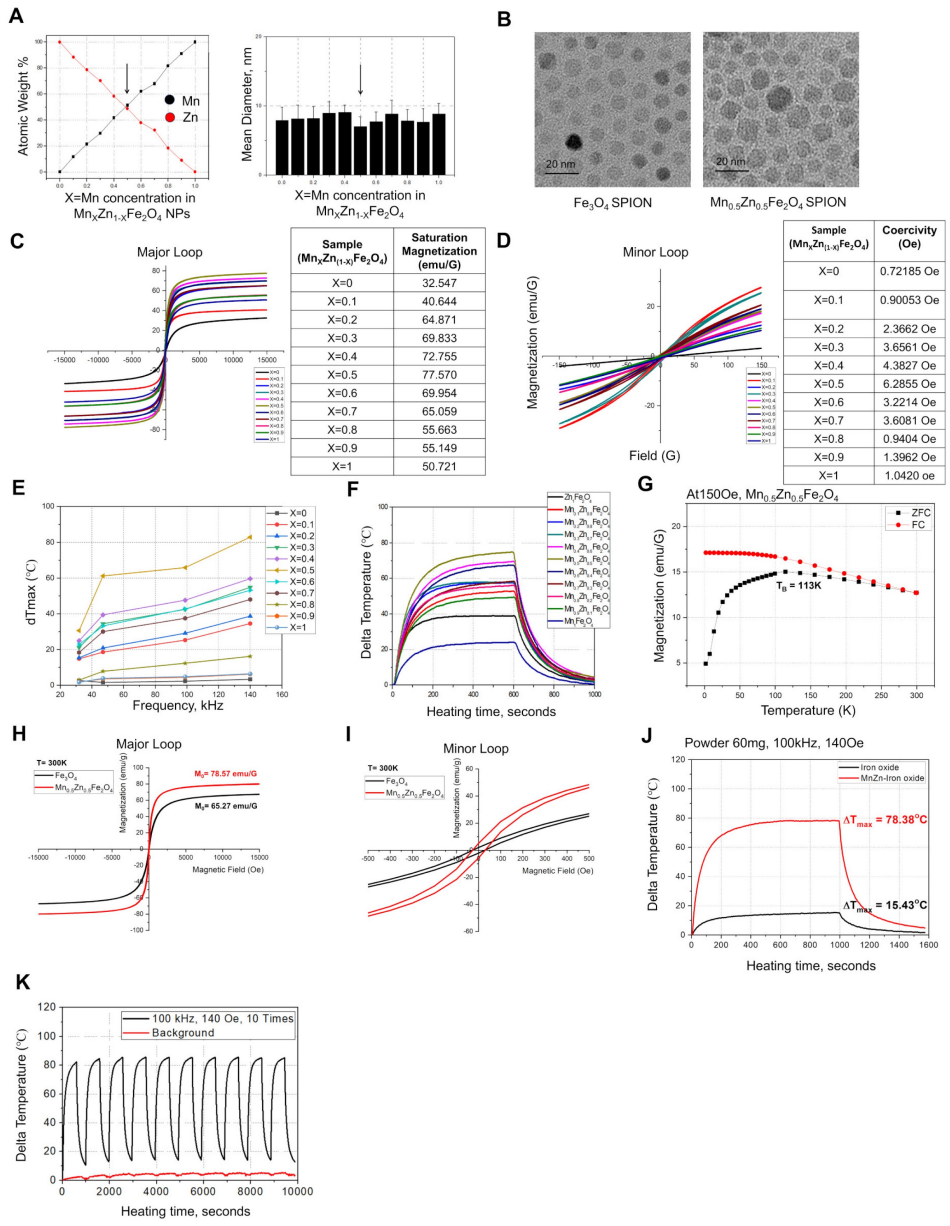
** Physical properties of Mn_x_Zn_1-x_Fe_2_O_4_ (_X=0~1, 1-X=1~0_) nanoparticles. A:** The mean diameter of the solid-state Mn_x_Zn_1-x_Fe_2_O_4_ superparamagnetic iron oxide nanoparticles (SPIONs) synthesized using the modified high-temperature thermal decomposition (mHTTD) method. **B:** transmission electron microscopy (TEM) image of the synthesized solid state Fe_3_O_4_ SPIONs (Fe_3_O_4_-SPION) (size: 6.5 nm ± 0.7 nm) and Mn_0.5_Zn_0.5_Fe_2_O_4_ SPIONs (MnZn-SPION-7) (size: 7.0 nm ± 1.5 nm) (magnified 100,000×). **C:** Major DC magnetization curves of the synthesized solid state Mn_x_Zn_1-x_Fe_2_O_4_ SPIONs, recorded at a field intensity of 15 kOe. **D:** Minor DC magnetization curves of the synthesized solid state Mn_x_Zn_1-x_Fe_2_O_4_ nanoparticles, recorded at a field intensity of 140 Oe. **E:** The AC heating characteristics of solid-state Mn_x_Zn_1-x_Fe_2_O_4_ nanoparticles under different frequencies of alternating currents (30, 50, 100, 140, 170, 200, 240, and 360 KHz) and a fixed magnetic field strength of 140 Oe. **F:** AC heating characteristics according to the composition of Mn and Zn in solid Mn_x_Zn_1-x_Fe_2_O_4(x=0~1, 1-x=1~0)_ nanoparticles at the fixed alternating current of 100 kHz and a magnetic field of 140 Oe. **G:** The ZFC/FC magnetization of MnZn-SPION-7 revealing a blocking temperature of 113 K. **H-I**: AC major loop of ZFC/FC magnetization with a maximum magnetization of 80 emu/g and a coercive field of 6.5 Oe observed in both major and minor hysteresis curves. **J:** Graph of changes in temperature of MnZn-SPION-7 (red) vs. Fe_3_O_4_-SPION (black) under an alternating magnetic field (AMF) at 100 kHz and 140 Oe for a duration of t = 1,000 s. Maximum temperature changes are labeled (78.38 °C vs. 15.43 °C). **K-L:** Graphs of MnZn-SPION-7 temperature change under repeated AMF exposures at 100 kHz AC and 140 Oe magnetic fields up to 10 times with or without background signals.

To determine the optimal Mn and Zn doping ratios, different ratios of Mn- and Zn-doped Fe_3_O_4_ NPs (Mn_x_Zn_1-x_Fe_2_O_4_ NPs, where x ranged from 0 to 1) were synthesized using mHTTD, a method used in our previous study (Figure S1A-N and Supplementary Tables 1, 2) 22. Among these synthesized variants, MnZn-SPION-7 exhibited the smallest size, measuring 7.0 ± 1.5 nm, which was comparable to the size of Fe_3_O_4_ SPION (Fe_3_O_4_-SPION), which measured 6.5 ± 0.7 nm (Figure 1A-B). Next, we compared the saturation magnetization values of all Mn_x_Zn_1-x_Fe_2_O_4_ particles. The major DC magnetization curves of all the variants, recorded at a field intensity of 15 kOe, revealed saturation magnetizations ranging from 32.5 emu/g to 77.6 emu/g, depending on the Mn and Zn compositions of the solid-state Mn_x_Zn_1-x_Fe_2_O_4_ NPs (Figure 1C). Minor DC magnetization curves recorded at a field intensity of 140 Oe revealed that all samples exhibited low coercive fields, typically between 0.7 and 6.3 Oe (less than 7 Oe), suggesting superparamagnetic behavior (Figure 1D).

To assess the magnetically-induced heating characteristics of solid-state Mn_x_Zn_1-x_Fe_2_O_4_ under alternating current (AC), the particles were subjected to AMF using a specially designed generation system at different frequencies and strengths (Figure 1E-G and Figure S2A-G). Among these NPs, MnZn-SPION-7 generated the highest heat when exposed to an AMF with a magnetic field of 140 Oe and an AC frequency of 100 kHz (H_appl_·f_appl_= 1.10 × 10^9^ A m^-1^s^-1^), which are safe for human use (Figure 1E-F). Additionally, we obtained the AC magnetization curves of various Mn_x_Zn_1-x_Fe_2_O_4_ NPs in the minor loop hysteresis under AMFs fixed under the same conditions (Figure S2H). The hysteresis loop area for solid-state MnZn-SPION-7 reached a maximum of 150, which was significantly larger than those of the other Mn_x_Zn_1-x_Fe_2_O_4_ NPs, indicating its superior energy dissipation and heat generation efficiency under AMF.

At room temperature, MnZn-SPION-7 exhibited superparamagnetic behavior, with a blocking temperature of 113 K (Figure 1G). Major hysteresis loop measurements at AC of ±15 kOe revealed the maximum magnetization values of 78.6 emu/g for MnZn-SPION-7 and 65.3 emu/g for Fe_3_O_4_-SPION (Figure 1H). In the minor AC magnetization curve, MnZn-SPION-7 had a coercive field of 6.3 Oe, whereas Fe_3_O_4_-SPION exhibited 2.5 Oe (Figure 1I). Notably, when exposed to an AMF of 140 Oe and an AC frequency of 100 kHz, 60 mg of MnZn-SPION-7 generated significantly higher heat than 60 mg of Fe_3_O_4_-SPION (78.4 °C vs. 15.4 °C) (Figure 1J). The calculated specific absorption rate (SAR) and intrinsic loss power (ILP) of MnZn-SPION-7 were five times higher than those for Fe_3_O_4_-SPION when exposed to an AMF of 140 Oe and an AC of 100 kHz (Figure S2I). To confirm long-term MnZn-SPION-7 stability, we repeatedly exposed the particles under the same conditions. Sixty mg of the synthesized MnZn-SPION-7 consistently demonstrated excellent AC heating characteristics (Figure 1K). These results suggest that the optimized Mn_x_Zn_1-x_Fe_2_O_4_ was likely MnZn-SPION-7.

### Structural properties of Mn_0.5_Zn_0.5_Fe_2_O_4_ SPIONs

**Figure 2:**
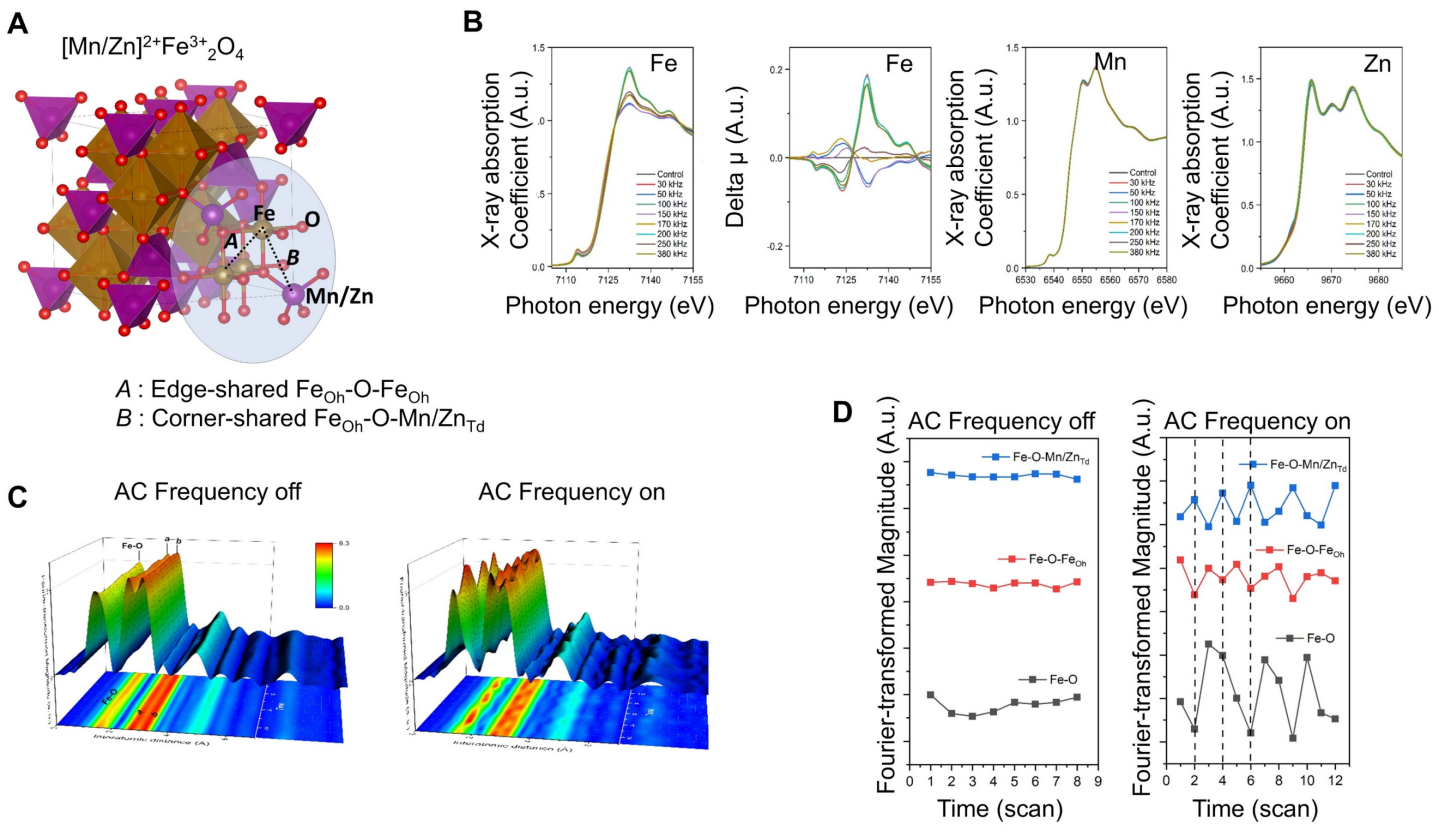
** X-ray diffraction (XRD) and X-ray absorption spectroscopy (XAS) of Mn_0.5_Zn_0.5_Fe_2_O_4_ SPION (MnZn-SPION-7) A:** 3D structure of the chemical oxidation state of iron (Fe) and the local coordination geometry within the spinel structure. **B:** Fe, Mn, and Zn K-edge X-ray absorption fine structures (XAFS) of the MnZn-SPION-7 under various AC magnetic fields (30, 50, 100, 150, 170, 200, 250, and 380 kHz). **C:** Spectral analysis of local structural Fourier-transformed radial distribution function (FT-RDF) around Fe ion with and without 100 kHz AC frequency and 140 Oe magnetic field. **D:** The FT magnitudes of Fe-O, Fe-O-Fe_Oh_, and Fe-O-[Fe/Mn/Zn]_Td_ bonds exhibiting an oscillating pattern of oxygen vacancy and site-migration between octahedral and tetrahedral sites in the spinel structure with an applied 100 kHz AC magnetic field.

To investigate the local structural variations in MnZn-SPION-7, Fe, Mn, and Zn K-edge X-ray absorption fine structures (XAFS) were determined at various AC frequencies (30, 50, 100, 150, 170, 200, 250, and 380 kHz) (Figure 2A-B). Notably, the valence states of Mn and Zn remain constant at +2 across all AC frequency variations, as evidenced by the consistent spectral features observed in the Mn and Zn K-edge XANES spectra. Conversely, the Fe K-edge XANES spectra changed markedly depending on the applied AC frequency, indicating the selective activity of the Fe site among the three transition metal ions in MnZn-SPION-7 with respect to different AC magnetic fields.

The XAFS feature variation arises from site redistribution around the Fe ions between the edge-shared Fe-O-Fe_Oh_ and Fe-O-[Fe/Mn/Zn]_Td_ bonds. These Fe-sensitive structural characteristics in response to AC magnetic fields were confirmed through long-term XAFS characterization under 100 kHz applied AC and a 140 Oe magnetic field. Indeed, a spectral comparison of the local structural Fourier-transformed radial distribution function (FT-RDF) around Fe ions with and without 100 kHz applied AC confirmed this (Figure 2C). When an AC magnetic field was applied, the FT peak intensities of Fe-O, Fe-O-Fe_Oh_, and Fe-O-[Fe/Mn/Zn]_Td_ bonding exhibited an oscillating pattern, indicating an effective variation in local structural environments around the central Fe atoms, such as the static disorder of each coordination with respect to alternating magnetic fields. Conversely, such FT peak oscillations were not observed under the DC magnetic field. (Figure 2D). These findings suggest that the activation of structural changes around the central Fe ions in the spinel structure under AC magnetic fields is a key factor in facilitating the hyperthermic reaction.

### *In vitro* application of PEGylated Mn_0.5_Zn_0.5_Fe_2_O_4_ SPIONs

**Figure 3:**
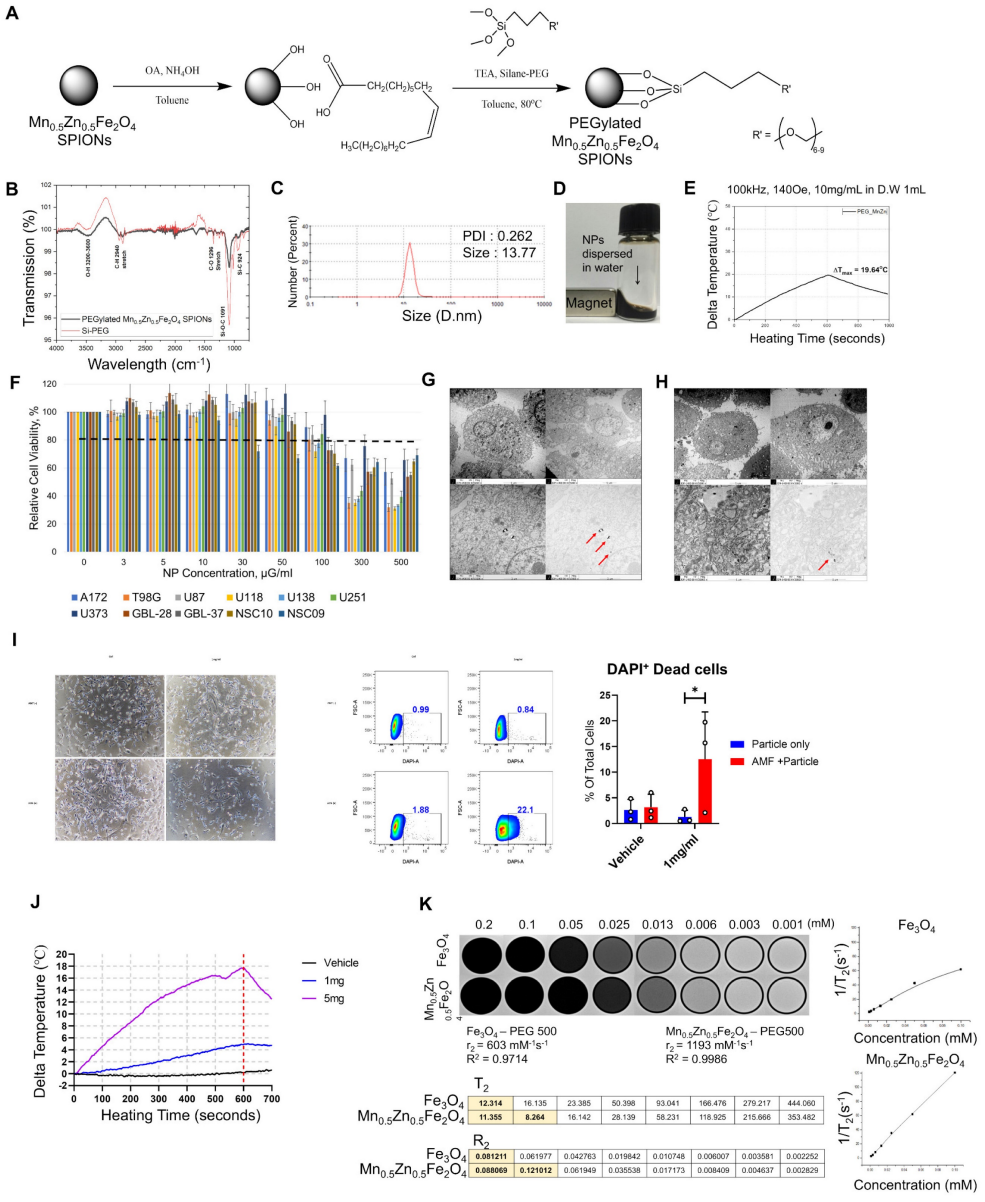
***In vitro* study of pegylated Mn_0.5_Zn_0.5_Fe_2_O_4_ SPION (PEG-MnZn-SPION-7). A:** A schematic diagram illustrating the surface modification of MnZn-SPION-7 with oleic acid and silane-PEG 500. **B:** the Fourier transformed infrared (FT-IR) spectra of pegylated MnZn-SPION-7 (PEG- MnZn-SPION-7) showing typical bands associated with C-H stretching, O-H stretching, and C-O stretching vibrations. **C:** Particle size graph determined by the Dynamic Light Scattering (DLS) of PEG-MnZn-SPION-7. The particle size was determined to be 13.77 nm and the polydispersity index (PDI) was 0.26. **D:** Demonstration of magnetism of PEG-MnZn-SPION-7 in an aqueous solution. **E:** Graph showing the changed temperature (19.64 °C) of 10 mg/mL PEG-MnZn-SPION-7 exposed to AMFs at a frequency of 100 kHz and a magnetic field strength of 140 Oe. **F:** *in vitro* cytotoxicity assay with different PEG-MnZn-SPION-7 concentrations on various cell lines of human glioblastoma and normal cortex. **G:** TEM images revealing internalized PEG-MnZn-SPION-7 by U87 cells. **H:** TEM images illustrating internalized PEG-MnZn-SPION-7 by NSC-10 cells. **I:** Representative images U87MG cells incubated with vehicle (Ctrl) or PEG-MnZn-SPION-7 (1 mg/mL), followed by 10 min of AMF treatment and 6 h incubation, along with flow cytometry scatter plot and bar graph showing DAPI-positive cells analyzed by flow cytometry. **J:** Temperature changes during AMF treatment of U87MG cultures treated with vehicle, 1 mg/mL, or 5 mg/mL PEG-MnZn-SPION-7. Temperature increases: Vehicle, 0.87 °C; 1 mg/mL, 4.77 °C; 5 mg/mL, 17.47 °C. **K:** T2 signal measurements of PEG-MnZn-SPION-7 using a 3 T MRI system in comparison with PEG-Fe_3_O_4_-SPION-7. *<0.05, multiple t- test.

To test the performance of MnZn-SPION-7 *in vitro,* PEGylated 7-nm Mn_0.5_Zn_0.5_Fe_2_O_4_ SPIONs (PEG-MnZn-SPION-7) were prepared (Figure 3A). PEG-MnZn-SPION-7 maintained a diameter of 13.77 nm with a polydispersity index (PDI) of 0.26 (Figure 3B, C). A simple test with a magnet demonstrated that the magnetic properties of PEG-MnZn-SPION-7 were maintained in the aqueous solution (Figure 3D). Upon exposure to an AMF at 100 kHz AC and a magnetic field of 140 Oe, the temperature of the PEG-MnZn-SPION-7 suspension increased by 19.6 °C at a concentration of 10 mg/mL (Figure 3E). We also confirmed that although the values of SAR and ILP decreased in comparison to the powder state, they remained at least three times higher than those for Fe_3_O_4_-SPION (Figure S2J). Next, we determined the cytotoxicity of PEG-MnZn-SPION-7s in nine glioblastoma cell lines and two primary cultured normal human cortex cells. Cell viability was tested using CCK-8 assay, and there were no viability changes with PEG-MnZn-SPION-7 treatment (<50 ug/ml) (Figure 3F). We also determined the particle distribution using transmission electron microscopy (TEM), which showed the internalization of SPIONs in all cell lines. PEG-MnZn-SPION-7s were localized in the cytoplasm without causing abnormal cell morphology (Figure 3G, H and Figure S4A-M).

After assessing the cytotoxicity and spatial localization of PEG-MnZn-SPION-7, we evaluated its anti-tumor activity *in vitro*. First, to ensure that PEG-MnZn-SPION-7 increased in temperature when infused with tumor cells, U87MG tumor cells (5 x10^6^ cells) were suspended in an aqueous solution containing 10 mg/mL SPIONs in an Eppendorf^Ⓡ^ tube. After exposing these tumor cells to AMF at 100 kHz and 140 Oe, the temperature increased by 26.6 °C, as shown in Figure S3A. Trypan blue staining revealed that the tumor cells were undetectable, indicating complete ablation due to the high-level heating. To confirm cell death in a cultured state, U87MG tumor cells (1 × 10⁵ cells/well) were treated with 1 mg/mL of PEG-MnZn-SPION-7 and analyzed for cell death using flow cytometry 6 h post-AMF treatment. The results showed a significant decrease in cell density after AMF treatment with 1 mg/mL SPIONs (Figure 3I). Flow cytometry confirmed the induction of cell death by AMF in the presence of PEG-MnZn-SPION-7. At 5 mg/mL, the temperature increased by 17.5 °C, whereas at 1 mg/mL, the temperature increased by 4.77 °C (Figure 3J). Given that typical hyperthermia strategies induce DNA damage within a temperature range of 39-41 °C, our PEG-MnZn-SPION-7 particles demonstrated effective hyperthermic properties for disrupting tumor growth.

Finally, we tested the diagnostic efficacy of PEG-MnZn-SPION-7 by measuring its T2 signals in a 3T MRI system (SIMENS, Berlin, Germany) and compared them with those of PEGylated Fe_3_O_4_ SPION (PEG-Fe_3_O_4_-SPION). Previously, it was reported that Mn and Zn ferrite influence the magnetization composition and magnetization peaks 23, 24. Because MnZn-SPION-7 exhibited superparamagnetic behavior, we wanted to confirm whether the T2 relaxivity (r2, mM^-1^s^-1^) also increased to determine if PEG-MnZn-SPION-7 is a better diagnostic tool for cancer. The T2 relaxivity of PEG-MnZn-SPION-7 was found to be 1193 mM^-1^s^-1^_,_ almost twice that of PEG-Fe_3_O_4_-SPION, which was only 603 mM^-1^s^-1^ (Figure 3K). Therefore, the *in vitro* test results confirm the high performance of PEG-MnZn-SPION-7 as a theranostic agent.

### *In vivo* application of PEGylated Mn_0.5_Zn_0.5_Fe_2_O_4_ SPION

**Figure 4:**
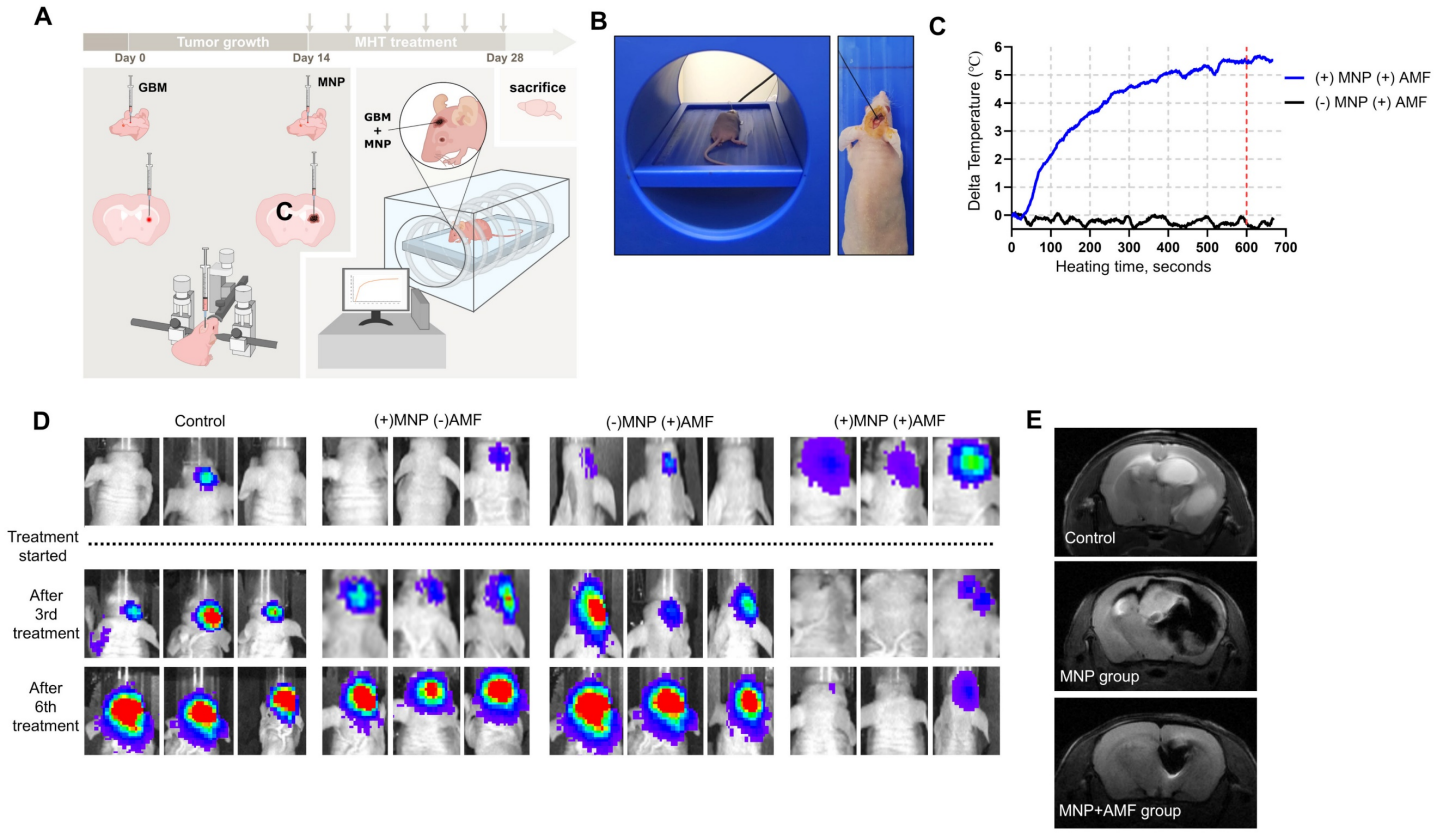
***In vivo* study of pegylated Mn_0.5_Zn_0.5_Fe_2_O_4_ SPION. A:** Schematic illustration of intratumoral application for magnetic hyperthermia treatment (MHT) in *in vivo* orthotopic nude mouse brain tumor models of U87MG expressing GFP and luciferase using 5 μL of PEG-MnZn-SPION-7 (30mg/mL). *C= contralateral* **B:** The intratumor core temperature monitoring setup consisting of a fiberoptic thermal sensor during MHT. **C:** Intratumoral temperature changes recorded for (+) MNP+AMF (Blue line) and (-) MNP+AMF (Black line) groups. The graph shows the temperature change over time (in seconds) during the first 10 min of MHT treatment, normalized against background temperature measured from the oral cavity. *Max Delta temperature value: Blue line=5.68°C, Black line=0.08°C*. **D:** IVIS images at 2 w after six cycles of magnetic hyperthermia treatment using 5 μL of PEG-MnZn-SPION-7 (30mg/mL) *in vivo* nude mouse orthotopic brain tumor models of U87MG expressing GFP & luciferase **E:** 9.4 T brain images (spin echo, TR: 2000 ms TE: 48 ms, FA: 90 ^o^, 1.0 mm thickness and gradient echo, TR: 2000 ms, TE: 5.2 ms, FA: 50^o^, 1.0 mm thickness) taken 1 w after three sessions of MHT using 5 μL of PEG-MnZn-SPION-7 (30 mg/mL).* n = 3* per group.

After confirming the anti-tumor potential of PEG-MnZn-SPION-7 and its high T2 relaxivity *in vitro*, we investigated MHT efficacy *in vivo* using an orthotopic xenograft brain tumor model in nude mice. Mice inoculated with U87MG tumor cells expressing GFP and luciferase were injected with PEG-MnZn-SPION-7 (30 mg/mL) two weeks after tumor growth (Figure 4A). Each MHT lasted 20 min per session, and six sessions were performed over a two-week period. The AMF parameters for each session were fixed at the highest heating conditions (100 kHz, 140 Oe) used in this study. To minimize the influence of background temperature increases caused by environmental factors, the temperature of the xenografted tumor was recorded for the first 10 min of each treatment session. The results showed an average temperature change of 5.68 °C (Figure 4B,C), which would be sufficient to induce mild hyperthermia.

An *in vivo* imaging system (IVIS) was utilized to visually observe tumor growth. Significant suppression of GBM growth was observed in orthotopic xenografts treated with MHT (MNP+AMF) compared to the control groups (Figure 4D). These results suggest that PEG-MnZn-SPION-7 particles combined with MHT effectively inhibited tumor progression, demonstrating their potential as a therapeutic agent for GBM treatment *in vivo*.

To confirm the diagnostic properties of PEG-MnZn-SPION-7, 9.4 T brain images (Agilent Technologies Inc., Santa Clara, USA) were obtained after three sessions of MHT (1-week post-treatment). Intratumoral injection of PEG-MnZn-SPION-7 resulted in a remarkably strong contrast enhancement of tumors, irrespective of their distribution as observed in the 9.4 T MRIs (Figure 4E). The images also revealed clear demarcations of orthotopic brain tumors; tumor sizes in the MNP+AMF group were significantly decreased, whereas those in the MNP group were notably increased, revealing tumor migration into the opposite hemisphere. Additionally, 3 T MRI (SIMENS, Berlin, Germany) demonstrated strong contrast enhancement of tumors after intratumoral or intravenous injection of PEG-MnZn-SPION-7 into the subcutaneous tumors of nude mice (Figure S5A). Furthermore, computed tomography (CT) showed contrast enhancement of the tumors following intratumoral or intravenous injection of PEG-MnZn-SPION-7 (Figure S5B), indicating the strong diagnostic capabilities of PEG-MnZn-SPION-7.

To determine whether PEG-MnZn-SPION-7 accumulated systematically, Prussian blue staining of various tissues, including the liver, kidney, spleen, lung, heart, muscle, brain, and eyeballs, was performed at different time points. The results showed accumulation primarily occurring in the liver and spleen, but clearance was observed 4 weeks post-treatment (Figure S6). No tissue damage was observed during the study. Thus, these findings suggest that MnZn-SPION-7 coated with PEG-500 serves as an efficient contrast agent for MRI, enabling effective cancer tracing.

### Elevation of Anti-tumor immunity by MHT with PEGylated Mn_0.5_Zn_0.5_Fe_2_O_4_ SPIONs

Previous studies have suggested that MHT upregulates anti-tumor immunity by enhancing both the innate and adaptive immune responses 7-14,25-30. Tumor cells undergoing immunogenic cell death (ICD) after various therapies, including hyperthermia, express calreticulin (CRT) on the cell surface and release tumor-specific antigens and “danger-associated molecular patterns” (DAMP), such as ATP, high mobility group box 1 protein I (HMGB1), type 1 interferons (IFNs), and heat-shock proteins (HSPs), thereby triggering a cascade of immune responses. DAMP and various immune cytokines, including IFN-૪, recruit and activate antigen-presenting cells (APCs), such as dendritic cells (DCs). Activated APCs phagocytize dying cells, migrate to nearby draining lymph nodes, and present tumor-specific antigens to naïve T cell, priming them into cytotoxic CD8^+^ T cells. These CD8^+^ T cells then migrate to the tumors and become activated. Additionally, cytokines released from dying tumor cells activate macrophages and NK cells, which, along with CD8^+^ T cells, kill tumor cells 25-32.

**Figure 5:**
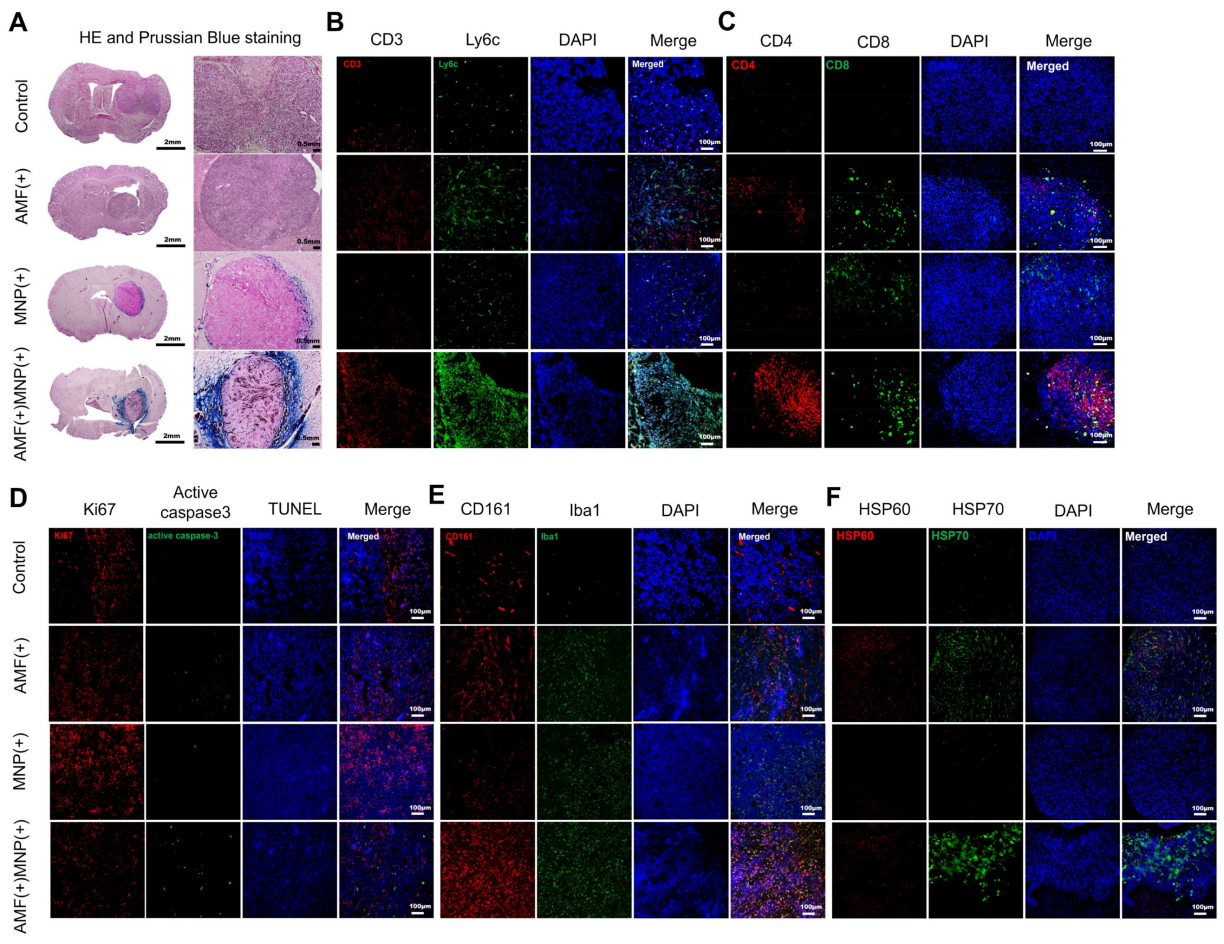
** H&E, Prussian Blue staining, and immunofluorescent staining of xenograft tumors. A:** The H&E and Prussian blue staining performed in the resected xenograft tumors of U87MG in the nude mouse models of four distinct groups. **B-F:** Immunofluorescent staining of different antibodies to view different targets as follows: **B:** CD-3 (T-cell), Ly6C (macrophage), **C:** CD4 (helper T-cell) and CD8 (cytotoxic T-cell). **D:** Ki67 (tumor proliferation), phosphorylated caspase-3 and TUNEL (apoptotic tumor). **E:** CD161 (NK cell) and Iba 1 (macrophage). **F:** HSP60 and HSP70 (heat shock protein signal). All images except **D** were achieved with DAPI Staining. *n = 5* per group.

To confirm whether MHT using PEG-MnZn-SPION-7 evoked significant anti-tumor immunity, we performed immunostaining in brain tumor mouse models bearing U87MG tumors 14 d after MHT treatment to evaluate the spatial distribution and retention of PEG-MnZn-SPION-7 within the tumor microenvironment. To investigate the temporal dynamics of NP localization, Prussian blue staining was performed at the early (2 d post-injection) and late (14 d post-treatment) stages of the MHT (Figure S7 and Figure 5A). These results confirmed that PEG-MnZn-SPION-7 initially penetrated the tumor core during the early phase of treatment and progressively accumulated at the tumor periphery in the later stage. We detected the infiltrating responses of CD3-positive (T-cell) and Ly6C-positive cells (monocyte-derived macrophages) within tumors in the AMF-MNP groups (Figure 5B). As nude mice have been shown to exhibit “leakage” in case of infection or inflammation 33, CD4 (helper T cell) and CD8 (cytotoxic T cell) were markedly increased in the AMF-MNP groups (Figure 5B, C). Moreover, the Ki-67 index (a cell proliferation marker) was significantly decreased in the MNP-AMF groups, while the expression of the apoptosis marker active caspase-3 and TUNEL staining were increased, suggesting reduced tumor cell proliferation and increased cell death (Figure 5D). Additionally, CD-161 (NK cells) and Iba1 (microglia) levels were markedly increased in the AMF-MNP group (Figure 5E). Finally, heat shock proteins (HSP 60, HSP70) were overexpressed in the AMF-MNP groups (Figure 5F), indicating their potential role in anti-tumor immunity, as described previously 34-44.

**Figure 6:**
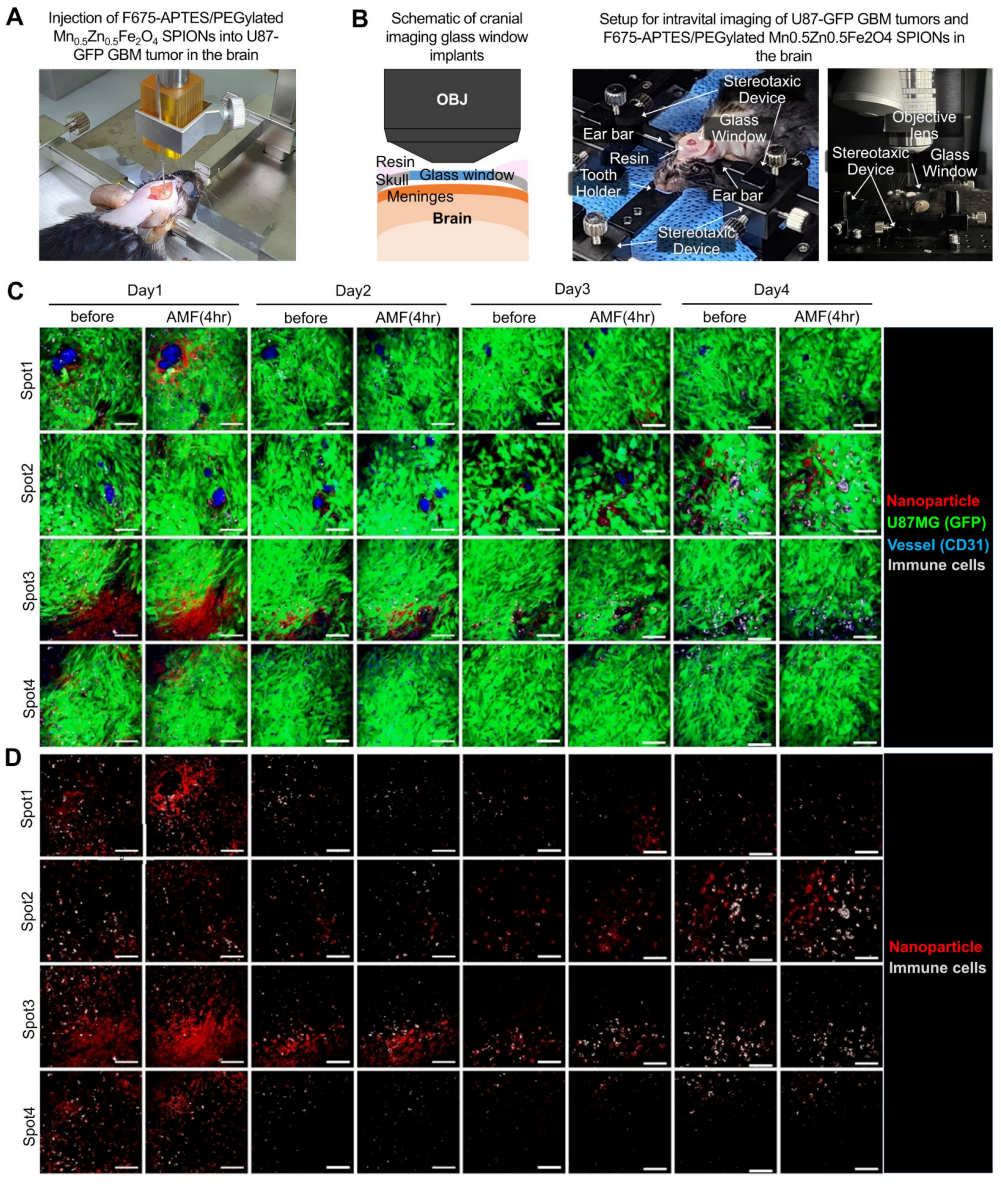
***In vivo* intravital microscopic visualization of immune cells and F675-APTES/ PEG-MnZn-SPION-7. A:** Stereotactic injection setup of F675-APTES/PEG-MnZn-SPION-7s. Particles were infused into mice bearing green fluorescent protein (GFP)-expressing U87 GBM tumors in the brain. **B:** Schematic of cranial imaging performed to monitor *in vivo* mobilization of immune cells into U87 GBM tumors in the subcortex of the mouse brain. A portion of the skull was removed and a cranial imaging glass window was implanted over the exposed brain area. Time-lapse imaging captured the fluorescence of GFP-expressing U87 GBM tumors and F675-APTES/PEG-MnZn-SPION-7s in the GBM infiltration zone. **C:** Monitored image for four different regions (Spot 1-4) of interest (ROIs) using an intravital fluorescence microscope at four different time points (day 1-4) post F675-APTES/PEG-MnZn-SPION-7s injections. Images were taken for 30 min immediately after MHT under an AMF of 140 Oe and 100 kHz AC every day, with a time interval of 24 h. **D:** Image from **C** without GFP signal for better visualization of particles and immune cells. Autofluorescence, which is expressed by immune cells, infiltrated the maximally dispersed spot of F675-APTES/PEG-MnZn-SPION-7s in the mouse brain, and the number of autofluorescent immune cells gradually increased over the course of day 1 to 4 just after MHT with F675-APTES/PEG-MnZn-SPION-7s under an AMF of 140 Oe at 100 kHz AC for 30 min each day.

To further investigate the anti-tumor immune response in tumors treated with PEG-MnZn-SPION-7 induced MHT, we utilized F675-APTES fluorescent-tagged PEG-MnZn-SPION-7 with U87MG expressing green fluorescence (GFP) grown in C57/Bl6 mice and performed time-lapse *in vivo* imaging using an intravital confocal microscope via the cranial window (Figure 6A,B and Figure S8). On day 1, the signals of F675-APTES/PEG-MnZn-SPION-7 were significantly increased near the perivascular area (Figure 6C, spot 1) or the GBM infiltration area (Figure 6C, spots 3 and 4) after MHT, with an AMF strength of 140 Oe and an AC frequency of 100 kHz for 30 min. Subsequent observations on days 2-4 with daily MHT application revealed strong autofluorescence, indicating highly phagocytic and endocytic macrophages serving as markers of cellular function 45, 46. The autofluorescent immune cells evidently infiltrated the areas with maximal dispersion of F675-APTES/PEG-MnZn-SPION-7 in the mouse brain from day 2 and remained there until day 4 (Figure 6C, spot 3; Figure 6D, spot 2,3). Interestingly, by day 4, an increase in both the F675-APTES/PEG-MnZn-SPION-7 signals and autofluorescent immune cell infiltration was observed.

To determine whether MHT treatment triggers the differentiation of immune cells into activated phenotypes, C57BL/6 mice splenocyte was co-cultured with a mouse glioblastoma cell line, GL261, expressing GFP. Splenocytes were co-cultured after the cell line culture was treated with PEG-MnZn-SPION-7 with or without AMF to avoid any extraneous variables. After two days, flow cytometry of the co-cultured cells revealed a significant increase in CD86^+^ macrophages in the MHT-treated group compared to that in the non-treated group (Figure S9A). Additionally, a large increase in the total CD45^+^ population and a significant increase in both CD4^+^ and CD8^+^ cells were detected, suggesting that MHT using PEG-MnZn-SPION-7 encourages immune recruitment, particularly activated phenotypes, further confirming what was observed *in vivo*, even within a short period (Figure S9B). These results suggest that MHT with PEG-MnZn-SPION-7 potentially modulates immune cell populations.

## Discussion

Over the past decades, patients with recurrent glioblastomas resistant to conventional therapies have undergone MHT with Fe_3_O_4_-SPION, usually in combination with radiation therapy 15, 47-49. Typically, 12-nm aminosilane-coated Fe_3_O_4_-SPIONs were injected into tumors for MHT, utilizing an AMF at a frequency of 100 kHz and varying magnetic field strengths ranging from 2.5-15 kA/m, achieving a maximum H_appl_·f_appl_= 1.5 × 10^9^ Am^-1^s^-1^. The concentration of Fe_3_O_4_-SPION was 112 mg/mL, and the median volume of magnetic fluid injected was 4.5 ml (ranging from 0.5-11.6 ml), equivalent to a median dosage of 0.28 ml of magnetic fluid per cm^3^ of tumor volume. Also, brain CT was used to estimate NP distribution in recurrent glioblastomas. This treatment improved the overall survival of the patients, suggesting Fe_3_O_4_-SPION as an effective cancer therapeutic strategy 49. Nevertheless, certain limitations such as non-tumoral off-target toxicity and low SAR values have hindered this magnetic NP to be used as a common therapeutic strategy 50-52. Hence, safer and more efficient NPs have been demanded through further research.

In this study, we designed MnZn-SPION-7 as a superparamagnetic NP optimized for hyperthermic applications with a minimal NP diameter to allow renal clearance. The performance of the NPs was evaluated in a physiologically safe AMF range (H_appl_·f_appl_ < 3-5 × 10^9^ Am^-1^s^-1^) 53, 54. Under these conditions, MnZn-SPION-7 achieved a SAR value of 10 W/g and an ILP value of 0.8 nHm²/kg 55. Therefore, we used ILP to accurately characterize and compare MnZn-SPION-7 with standard SPIONs owing to its heating ability. The typical ILP value was 0.15 nHm^2^/kg for standard SPIONS and 0.7nHm^2^/kg for Mn- and Zn-doped SPIONs 22, 55, 56. Our MnZn-SPION-7, with an ILP of 0.8 nHm²/kg, demonstrates superior energy dissipation and heating efficiency. This is particularly notable, given the particle size of 7 nm, which is smaller than the typical range for commercial SPIONs (10-20 nm). The smaller size allows for enhanced biocompatibility and renal clearance without compromising MHT performance.

In terms of diagnostic ability, intratumoral injections of PEG-MnZn-SPION-7 demonstrated remarkable efficacy in tracing glioblastomas, leveraging their pronounced T2 shortening effect on both 9.4 T and 3 T MRIs. Furthermore, our particles demonstrated remarkably good T2 relaxivity value compared to previous studies. Some studies have reported T2 relaxivity values of 385 mM^-1^s^-1^ for 14-nm core SPIONs coated with PEG1000 and 995 mM^-1^s^-1^ for 5-nm hydrophobic IONPs in bilayer hydrophilic MNPs, which represent the highest relaxivity per Fe atom 57, 58. Meanwhile, the T2 relaxivity result shown by PEG-MnZn-SPION-7 was as high as 1193 mM^-1^s^-1^. To the best of our knowledge, the T2 relaxivity of PEG-MnZn-SPION-7 coated with PEG-500 observed in this study is the highest T2 relaxivity value reported for SPIONs, confirming its potential diagnostic efficiency. Therefore, PEG-MnZn-SPION-7 is a highly promising theranostic platform, uniquely tailored for precise and synergistic applications of MRI and MHT under AMF, and holds significant potential for tracing and treating various cancers, including glioblastomas.

Our *in vivo* and *in vitro* experiments demonstrated the capability of MnZn-SPION-7 to effectively elevate the temperature of SPION suspensions when exposed to an AMF, demonstrating its potential for use in MHT. Surface modification with methoxy-PEG-silane optimizes its biological functionality and safety. It should be noted that the PEG-MnZn-SPION-7 reported in this study may have a lower value than other studies applying magnetic hyperthermia such as He* et al.* 64 and Hazarika* et al.* 65. However, these NPs required higher magnetic field parameters (> 300 kHz) than our physiologically safe parameters (AMF at 100 kHz, MF of 140 Oe). If tested at a stronger magnetic field intensity, MnZn-SPION-7 would have had comparably higher values.

Moreover, we simulated the application of MHT with PEG-MnZn-SPION-7 on U87MG glioblastomas in athymic nude mice and demonstrated a decrease in tumor size along with a moderate increase in temperature of approximately 5.7 °C. Such parameters are sufficient for mild hyperthermia, but not for tumor ablation, which requires increased temperatures of >10 °C (above 46 °C). While this may seem that our particle is not efficient for therapeutics, it must be highlighted that only 5 μL of PEG-MnZn-SPION-7 with a concentration of 30 mg/ml (equivalent to 0.15 mg of PEG-MnZn-SPION-7) was injected into tumors at a safe but low intensity of AMF (H_appl_·f_appl_=1.10 × 10^9^ Am^-1^s^-1^). Additionally, it is important to note that tumor temperatures were measured by inserting thermal probes into the core of tumors, reflecting the intratumor temperatures raised by our heated SPIONs rather than the temperature of the SPIONs themselves. Moreover, the temperature of PEG-MnZn-SPION-7 was markedly higher than the measured intratumor temperature, and the sites inside the cells where the heated SPIONs were lodged led to cancer cell death. Also, the median amount injected in the present study was equivalent to 0.18 ml of PEG-MnZn-SPION-7 per cm^3^ of tumor volume. These conditions are biologically and physiologically safer for humans than MHT using 12-nm aminosilane-coated Fe_3_O_4_-SPION, as previously reported 15, 47-49. Therefore, MnZn-SPION-7 can achieve anticancer activity in very small amounts, making it a highly efficient SPION.

In addition to the increased temperature data, we also obtained data supporting the activation of immune cells. Together with additional *in vitro* data that determined an increase in T cells and CD68^+^ macrophages, our results demonstrate the therapeutic potential of our particle-inducing anti-tumor immune cells. The observed infiltration of macrophages and NK cells into glioblastoma tissues, accompanied by the overexpression of HSPs, suggests a dual mechanism of action that combines heat-induced cellular damage with an increase in anti-tumor immunity. Hyperthermia can activate the immune system through different mechanisms such as inducing antigenicity or suppressing anti-inflammatory signaling 59, 60. Heat shock protein-dependent activation is well known for enhancing the effect of hyperthermia in tumors 61-63. Investigating how the MnZn-SPION-7 induced MHT impacts the overall immune system *in vivo* is intriguing.

In summary, MnZn-SPION-7 not only outperformed standard SPIONs in terms of heating efficiency but also provided advantages in terms of safety and clinical applicability. Its ability to achieve effective hyperthermia at lower doses and under safer AMF conditions underscores its potential for future applications in MHT, which we believe will be a milestone in the development of therapeutic agents for advanced MRI and MHT.

## Materials and methods

### Preparation of superparamagnetic iron oxide nanoparticles (SPIONs)

A modified high-temperature thermal decomposition method (mHTTD) was used in this study 19. Modifications were made with the ramping-up rate and the heat treatment time from the conventional HTTD method 64. The ramping-up rate used was 1.2 °C/min and the heat treatment time was 38 min to manipulate the Mn cation concentration and particle dipole interaction in all ferrite sites. Figures 1A and B show the mHTTD process and Supplementary Table 1 lists elemental amounts. Fe(III) acetylacetonate (> 99.9%), Mn(II) acetate tetrahydrate (99.99%), Zn(II) acetate dihydrate (99.999%), oleic acid (90%), oleylamine (70%), and benzyl ether (99%) were purchased from Aldrich Chemical Co., and1,2-hexadecanediol (>98%) was purchased from Tokyo Chemical Industries Co. (Supplementary Table 1).

For the synthesis of Fe_3_O_4_ and manganese and zinc-doped iron oxide NPs (Mn_x_Zn_1-x_Fe_2_O_4_, where x ranges from 0 to 1) using the mHTTD method; Mn(II) (0.5 mmol), Zn (0.5 mmol), Fe(III) (2 mmol), oleic acid (6 mmol), oleylamine (6 mmol), 1.2 hexadecanediol (10 mmol), and benzyl ether (20 mL) were mixed in a three-neck flask and magnetically stirred at room temperature (as shown in Figure S1A). Initially, the reaction solutions were heated to approximately 200 °C from room temperature at a rate of approximately 9 °C/min (the initial ramping rate) and maintained for 60 min to have the nucleation process during the heat treatment time at 200 °C. Subsequently, the reaction solutions were heated to approximately 300 °C for 10 min (at a rate of approximately 10 °C/min, the second ramping rate) and maintained for 50 min for the growth process during heat treatment at 300 °C (Figure S1B). Next, the mixed solution was cooled to room temperature (approximately 20 °C), and ethanol (40mL) was added to rinse the synthesized NPs under ambient conditions. The synthesized products, which exhibited a black or brown color depending on the Mn and Zn doping ratios, were rinsed, precipitated, and separated using a centrifuge. The products were then dissolved in hexane (15 mL), mixed with ethanol (30 mL), and magnetically stirred for an additional 30 min to remove organic residues. After centrifugation at 6500 rpm for 12.5 min, the NPs were collected and dried at room temperature.

### Vibrating sample magnetometer

A vibrating sample magnetometer (VSM; Toei Kogyo, VSM-5) was used to test the magnetic properties of the synthesized Fe_3_O_4_ and Mn_x_Zn_1-x_Fe_2_O_4_ NPs. Both the major and minor magnetic hysteresis loops and initial magnetization curves were obtained. The applied magnetic fields were ±15 kOe for major hysteresis loops, and ±140 Oe for minor hysteresis loops. DC magnetization curves were measured at room temperature using the VSM equipped with an electromagnet (TEM-WFR7, Toei Kogyo) and a Gaussian meter (Model 421, Lake Shore Cryotronics, Inc.). AC magnetization curves were also measured within the frequency range of 30-360 kHz under applied field amplitudes of 80-160 Oe using a specially designed AC magnetization device equipped with a 210-turn water-cooled solenoid coil with a diameter of 16.0 mm for excitation (Figure S2A,B). For each measurement, 15-20 mg of solid-state Fe_3_O_4_ and Mn_x_Zn_1-x_Fe_2_O_4_ NPs were placed in a VSM holder at room temperature.

### Measurement of AC magnetically generated heating characteristics

The AC magnetically induced heating temperatures of the solid-state and coated Mn_x_Zn_1-x_Fe_2_O_4_ NPs were measured using the specially designed AC magnetic field generation system described above. The total quantity of solid-state nanoparticles used to measure the AC heating characteristics was fixed at 60 mg. To experimentally determine the specific loss power (SLP), the coated NPs were dispersed in various viscous fluids at different concentrations, and the AC heating temperatures of the fluids containing the synthesized Fe_3_O_4_ and Mn_x_Zn_1-x_Fe_2_O_4_ NPs were measured under the applied AMFs. AMF was applied for the initial 600 s, and the heating temperatures were recorded using an optical thermometer for up to 1,000 sec. To investigate their hyperthermic characteristics, solid-state and PEGylated Fe_3_O_4_ and Mn_x_Zn_1-x_Fe_2_O_4_ NPs were placed in Eppendorf tubes and measured using an AMF generation system. Eppendorf tubes containing solid-state Fe_3_O_4_ and Mn_x_Zn_1-x_Fe_2_O_4_ NPs were insulated with Styrofoam to minimize environmental influences. The tip of the optical fiber used for temperature measurement was positioned inside an Eppendorf tube containing solid-state Fe_3_O_4_ and Mn_x_Zn_1-x_Fe_2_O_4_ NPs. After the temperature measurement, the SAR and ILP of MnZn-SPION-7 and Fe_3_O_4_-SPION were calculated using the following equations:


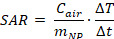


C*_air_ =* air capacity (0.24kcal/kg°C), M*_NP_* = Particle mass, *∆T* = Temperature change, *∆t* = Total Time change,



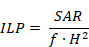



f = Frequency (Hz), H = Henry (Oe).

### XAFS analysis of synthesized Mn_x0.5_Zn_1-x_Fe_2_O_4_ nanoparticles

Using the BL10C beam line at the Pohang light source (PLS-II) with top-up mode operation under a ring current of 250 mA at 3.0 GeV, the Fe, Mn, and Zn K-edge X-ray absorption spectra of the synthesized 7-nm Mn_x0.5_Zn_1-x_Fe_2_O_4_ NPs exposed to various AC frequencies (30, 50, 100, 150, 170, 200, 250, and 380 kHz), X-ray absorption near edge structure (XANES) and extended X-ray absorption fine structure (EXAFS) were collected. A monochromatic X-ray beam was obtained using a liquid-Ni-cooled Si double-crystal monochromator (Bruker ASC) from the high-intensity X-ray photons of a multipole wiggler source. All X-ray absorption spectroscopic data were recorded in the transmittance mode using an ionization chamber array (IC-SPEC, Oxford Ltd.) as a photon detector. Higher-order harmonic contamination was eliminated by detuning to reduce the incident X-ray intensity by approximately 30%. Energy calibration was simultaneously performed for each measurement, with each reference metal placed in front of the third ion chamber. Additionally, long-term structural variation around Fe ions for the 7-nm Mn_0.5_Zn_0.5_Fe_2_O_4_ superparamagnetic iron oxide (SPION) (MnZn-SPION-7) was measured under a 100 kHz AC and 140 Oe magnetic field for 6 h.

### Surface modification of synthesized SPIONs

The synthesized Fe_3_O_4_-SPION and 7-nm MnZn-SPION-7 were coated with biocompatible methoxy-PEG-silane with a molecular weight of 500 Da. Methoxy-PEG-silane 500 Da (Gelest Inc.), and trimethylamine were obtained from Sigma-Aldrich. To establish the PEG coating, the surfaces of the synthesized NPs were initially modified with oleic acid. Specifically, 3 mL of oleic acid and 0.7 mL NH_4_Cl were added to an ethanol solution (30 mL) containing the NPs. The mixture was agitated vigorously for 2 h and washed with acetone. Afterwards, the NPs were precipitated using a permanent magnet, resulting in oleic acid-coated NPs. Next, the oleic-acid-modified NPs were dispersed in toluene (7.5 mL), followed by (3.75 mL) triethylamine and 0.75 mL of methoxy-PEG-silane 500 Da. The mixture was agitated thoroughly for 24 h. PEG-coated Fe_3_O_4_ (PEG-Fe_3_O_4_-SPION) and Mn_0.5_Zn_0.5_Fe_2_O_4_ SPIONs (PEG-MnZn-SPION-7) were washed with pentane and dispersed in water to create nanofluidic solutions, as shown in Figure 3D.

### Measurement of the relaxivity of contrast agents

To compare the magnetic properties of PEG-MnZn-SPION-7 with PEG-Fe_3_O_4_-SPION, T2 relaxivity was measured using a 3 T MRI system (SIMENS, Berlin, Germany). T2-weighted multi-echo spin-echo sequence was acquired using the following parameters: TR = 3000 ms; TE = 10 ms; number of echoes=32; flip angle (FA) = 180 ^o^; field of view (FOV) = 179.2 × 89.6 mm^2^; pixel size = 0.56 × 0.56 mm^2^; slice thickness = 5 mm. Two to eight wells were prepared using eight different concentrations of solutions (0.001, 0.003, 0.006, 0.013, 0.025, 0.05, 0.1, and 0.2 mmol) for each type of SPION. The measured data from the system were graphed via the two-parameter fitting method in MATLAB, as described by Rohrer *et al.* 65.

Regions of interest (ROIs) were manually selected within the well, and the averaged values within the ROIs were obtained with 32 TEs from 10 to 320 ms. The T2 relaxation time was measured by fitting an exponential model to the average values. The R2 relaxation rate, 1/T2, was plotted as a function of the contrast agent concentration, and the linear parameter, relaxivity, was estimated using the least squares method.

### Dynamic light scattering

The hydrodynamic diameter (dH), distribution, polydispersity index (PDI), and Z-potential of the ferrofluids with silica- and PEG-coated NPs were measured using a dynamic light scattering system (DLS, Zetasizer Nano ZS System). We also determined the dispersion, aggregation, and surface charge of MnZn-SPION-7, which influenced its magnetic properties, AC magnetically induced heating characteristics, and cellular uptake efficiency.

### Cell culture

We used 11 different cell lines: human brain cortex cells (NSC09 and NSC10), primary human glioblastoma cells (GBL-28 and GBL-37), and glioblastoma cells (A172, T98G, U118, U138, U251, U373, and U87). Primary human cell lines were obtained with Institutional Review Board (IRB) approval from Seoul National University Hospital (IRB No.H-0507-509-153). Commercially available human glioblastoma cell lines were obtained from the American Type Culture Collection (ATCC; Manassas, VA, USA) and Korean Cell Line Bank (KCLB: Seoul, Korea). All cells were cultured with Dulbecco Modified Eagle Medium (DMEM, WelGENE, LM001-05, Korea) containing 10% fetal bovine serum (FBS, Gibco Corp., 16000, Grand Island, NY, USA) and 100 U/ml penicillin/streptomycin (Gibco Corp., 15140-122, Grand Island, NY, USA) in a humidified incubator with 5% CO_2_ at 37 °C and accessed by trypsinization using TrypLE™(Gibco Corp., 12604-013, Grand Island, NY, USA) every 7-8 d.

### *In vitro* cytotoxicity assay

The CCK-8 assay (WST-8; Dojindolabs, Kumamoto, Japan) was performed according to the manufacturer's instructions. All cultured cell lines were seeded at a density of 3000 cells/well in a 96-well plate. After 24 h, PEG-MnZn-SPION-7 was added at various concentrations (0, 3, 5, 10, 30, 50, 100, 300, and 500μg/mL) and incubated for 24 h in a humidified incubator at 37 °C with 5% CO_2_. Each well was then washed with phosphate-buffered saline (PBS), and fresh medium with CCK-8 solution was added to each well of the 96-well plate and incubated for another 2 h. Finally, the absorbance at 450 nm was evaluated using a Multiscan MS spectrophotometer (Lab Systems, Stockholm, Sweden).

### Flow cytometry for cell death rate and splenocyte co-culture assay

GL-261-GFP or U87MG GBM cells were cultured in a 24 well cell culture plate (SPL) at 1×10^5^/well density for 24 h. For the cell viability test, U87MG GBM cells were given 5 mg/ml and 1 mg/ml concentrations of PEG-MnZn-SPION-7s along with vehicle (1% DMSO in cell culture media). Each experiment was performed in triplicate. Cells were given either AMF for 600 s or were incubated in a humidified incubator at 37 °C with 5% CO_2_. After AMF treatment, all cells were washed with 37-°C 1X PBS twice to remove excess particles and fresh cell culture media was replenished. Cells were incubated for 6 h, then through a 70 μm cell strainer in a FACS tube with FACS buffer (5% FBS in PBS). Five min prior to flow cytometry, DAPI (Invitrogen, Carlsbad, CA, USA, 62248) was added at a ratio of 1:5000 for live/dead cell detection.

For the splenocyte co-culture assay, cells were administered vehicles or 2 mg/ml PEG-MnZn-SPION-7s and were compared with or without AMF, as mentioned above. Splenocytes were derived from 12-week-old C57/BL6 mice and seeded at a density of 1.5×10^5^/well. Splenocytes were not exposed to AMF to exclude the confounding variable of AMF affecting their differentiation. Co-culture was incubated for 48 h before staining with the following antibody cocktails 1:500 (diluted in FACS buffer): CD45 BUV395, CD11b V450, CD64 APC, F4/80 PE, CD3 BV 711, CD 4 BV 786, CD 8 APC-cy7, CD206 BV605, MHC II BV786, CD86 BV 711, and CD11c BV510 (all antibodies were purchased from BioLegend). The co-culture was incubated in the antibody cocktail for 20 min, then centrifuged at 2000 rpm for 5 min, resuspended in FACS buffer (200 μl), and was moved to a FACS tube before flow cytometry data acquisition in FACS symphony A5 (BDscience). All data were gated and analyzed using FlowJov10.10.

### Transmission electron microscopy (TEM) imaging in cells

TEM was conducted on cells treated with PEG-MnZn-SPION-7 to investigate the extent of NP penetration into the cells, cell apoptosis, and cell deformation, including nuclear fragmentation. The cells were incubated with 100 μg/mL PEG-MnZn-SPION-7 for 24 h. After incubation, cells were washed and detached using trypsin. Subsequently, cells were fixed overnight in a mixture of cold 2.5% glutaraldehyde in 0.1 M phosphate buffer (pH 7.2) and 2% paraformaldehyde in 0.1 M phosphate or cacodylate buffer (pH 7.2). Cells were then post-fixed for 1.5 h in 2% osmium tetroxide in 0.1 M phosphate or cacodylate buffer at room temperature. The samples were briefly washed with deuterated H_2_O_2_, dehydrated through a graded series of 50, 60, 70, 80, 90, 95, and 100% ethanol (x2), infiltrated with propylene oxide and EPON epoxy resin mixture (Embed 812, Nadic methyl anhydride, poly Bed 812, dodecenylsuccinic anhydride, dimethylaminomethyl phenol; Electron Microscopy Polysciences, USA), and finally embedded in epoxy resin only. The epoxy resin-mixed samples were loaded into capsules and polymerized at 37 °C for 12 h and 60 °C for 48 h. Sections for light microscopy were cut at 500 nm and stained with 1% toluidine blue for 45 s on a hot plate at 80 °C. Thin sections were prepared using an ultramicrotome (RMC MT-XL) and placed on a copper grid. Appropriate areas were cut to a thickness of 65 nm and stained with saturated 6% uranyl acetate and 4% lead citrate before TEM examination (JEM-1400; Japan) at 80 kV.

### *In vivo* animal tumor model using nude mouse

All animal experiments and care were performed according to the guidelines approved by the Institutional Animal Care and Use Committee (IACUC) of Seoul National University (IACUC No. SNU-161129-2). Mice were housed in an animal care facility, under 12-h light/dark cycle with access to food and water. All possible measures were taken to minimize suffering and to reduce the number of animals used in this experiment. All mice used for modeling were adult male BALB/c nude mice 10-12 weeks old. *In vivo* MHT was performed using U87MG tumors grown in the brains of adult male BALB/c nude mice. For intracranial mouse tumor models, mice were anesthetized, and U87MG cells expressing GFP and luciferase (3×10^5^ cells with volume of 5 μL) were injected into the brains of nude mice weighing 20-22 g using a Kopf animal stereotactic frame (David Koph Instruments, Tujunga, USA) (orthotopic xenograft brain tumor model) (Figure 4A). A craniectomy approximately 2-3 mm in diameter was performed using an electrical drill. The dura mater was punctured using a 30 G needle (approximately 0.3 mm). A Hamilton syringe connected to a 23 G, 45 ° needle was mounted on a syringe pump connected to a stereotaxic device. U87MG cells expressing GFP and luciferase (3×10^5^ cells with volume of 5 μL) were injected at coordinates 3 mm lateral to bregma and approximately -3.5 mm inferior to the skull at a speed of 0.5 µL per minute. U87MG cells expressing GFP and luciferase were obtained from Dr. Yoon's Laboratory at the Department of Nuclear Medicine, Seoul National University Hospital.

For subcutaneous mouse tumor models, mice were anesthetized, and FSaII (fibrosarcoma of C3H mice) cells (5×10^6^ cells with volume of 50 μL) were subcutaneously injected into the proximal thigh region of BALB/c nude mice. FSaII cells were obtained from Dr. C. W. Song at the University of Minnesota.

### *In vivo* magnetic nanoparticle hyperthermia

Two weeks after the injection of U87MG cells into the brains of nude mice, intracranial tumor mice models were anesthetized, and 5 μL PEG-MnZn-SPION-7 at a concentration of 30 mg/mL was injected into the center of the tumors. Each mouse was positioned at the center of the AC coil system for magnetic hyperthermia. An applied magnetic field strength of 140 Oe and a frequency of 100 kHz were used (equivalent to 11.14 kA/m with a total field dose of 1.10 x 10^9^ Am^-1^s^-1^). The hyperthermia treatment involved applying an AC magnetic field for 20 min, which was repeated six times over two weeks. The core temperature of the brain tumor was measured using an intracranial fiber optic sensor and an optical thermometer (Luxtron M600 & M924, LumaSense Technologies, Ballerup, Denmark).

### CT

The subcutaneous mice tumor model described above was used for computed tomography (CT). Two weeks after tumor injection, CT scans were conducted using a preclinical PET/CT scanner (SuperArgus 4r, Sedecal, Madrid, Spain). Images were captured before and after the administration of PEG-MnZn-SPION-7. These scans were conducted at 10 min, 1 h, and 4 h after either intratumoral or intravenous injection of the NPs.

### 3.0 T MRI

Two weeks after subcutaneous injection, the subcutaneous mouse tumor model was subjected to 3 T MRI. Images were captured before and after the administration of PEG-MnZn-SPION-7. Scans were conducted at 10 min, 1 h, 4 h, and 2 d after either intratumoral or intravenous injection of the NPs. 3.0 T MRI was conducted on a 3 T MRI system with a 6-channel animal coil (SIMENS, Berlin, Germany). Images were collected using T2-weighted turbo spin-echo sequences for each mouse with the following parameters: repetition time (TR) = 3000 ms; echo time (TE) 100 ms; field of view (FOV), 35 × 35 mm^2^; and slice thickness = 0.8 mm.

### 9.4 T brain MRI

One week after three sessions of MHT using 5 μL of PEG-MnZn-SPION-7 at a concentration of 30 mg/mL, brain images were obtained from intracranial mouse tumor models using a high-field 9.4T MRI system (Agilent Technology Inc, Santa Clara, USA). Images were obtained for the control, magnetic NP (MNP) group, and MNP+AMF (magnetic nanoparticles with alternating magnetic field) groups. Two pulse sequences were used. First, a T2-weighted turbo spin-echo sequence was applied with the following sequence parameters: TR = 2000 ms, TE = 48 ms, FA = 90 ^o^, and slice thickness = 1.0 mm. Additionally, a T2^*^-weighted gradient echo sequence was employed to capture images with the following settings: TR = 2000 ms; TE = 5.2 ms; FA = 50 ^o^; slice thickness = 1.0 mm.

### H&E staining

All tissues harvested from mice were fixed in 10% formalin at room temperature. The formalin-fixed tissues were then dehydrated, washed in ethanol, and embedded in paraffin. The brain tissues were sectioned into 10-μm thick segments and other tissues were sectioned into 4-5 μm. Sections were stained with H&E using an H&E staining kit (Abcam, USA). Stained sections were observed using a bright-field microscope (Nikon Co., Tokyo, Japan).

### Prussian blue staining

A Fe staining kit (Abcam, ab150674, Cambridge, UK) was used to determine the presence of Fe in the tissues. A solution consisting of 2% hydrochloric acid and potassium ferrocyanide was prepared by mixing them in a 1:1 ratio, and tissue slides were incubated with this solution for 20 min. Following incubation, the slides were washed with triple distilled water and subjected to nuclear fast red staining. Images of stained cells were captured using a BX43 light microscope (Olympus, Tokyo, Japan).

### Immunofluorescence staining

The brain tissue was fixed in 4% paraformaldehyde. After fixation, it was embedded in paraffin and then sectioned into 4 µm-thick segments on glass slides. Antigen epitope retrieval was achieved via heat induction. Tissue slides were blocked using 1X PBS containing 3% normal serum, followed by incubation overnight at 4 °C with different primary antibodies with a listed dilution rate as follows: anti-Ki67 (Dako, M7240, clone MIB-1; dilution, 1:100-200), anti-active caspase-3 (BD Pharmingen, 559565, clone C92-605; dilution, 1:1000-1500), *In Situ* Cell Death Detection Kit, Fluorescein (Roche, 11684795910; dilution, 1:200), anti-CD-45RA (Sigma-Aldrich, 05-1413, clone MEM 56; dilution, 1:100), anti-CD-138 (Avivasysbio, OABF00623; dilution, 1:100), anti-CD-3 (Abcam, USA, ab5690; dilution, 1:100), anti-CD-4 (GeneTex, GTX50984; dilution, 1:50), anti-CD-8 alpha (GeneTex, GTX41819, clone YTS105.18; dilution, 1:200), anti-Ly6C (Invitrogen, PA5-119794; dilution, 1:100), anti-CD-161 (Invitrogen, MA1-70100, dilution, 1:100), anti-Iba1 (Abcam, ab153696; dilution, 1:100), anti-HSP60 (Invitrogen, PA5-34760; dilution, 1:500), and anti-HSP70 (Invitrogen, MA3006, clone 3A3; dilution, 1:100). Subsequently, the slides were washed with 1X PBS three times, followed by incubation with secondary antibodies (Alexa Fluor 488, Invitrogen, A21202; Alexa Flour 594, and Invitrogen, A11032). Applied secondary antibodies were chosen based on the primary antibodies used (anti-mouse monoclonal for Ki67(AF594), CD-45RA (AF594), CD-161 (AF594), and HSP70 (AF488); anti-rabbit polyclonal for CD-3 (AF594), CD-4 (AF594), CD-138 (AF488), active caspase-3 (AF488), Ly6C (AF488), Iba1 (AF488), HSP60 (AF594); and anti-rat monoclonal for CD-8 alpha (AF488)). Antigenic signals were generated according to the manufacturer's instructions. Finally, the nuclei were counterstained with DAPI and mounted using a Permount Mounting Medium (Thermo Fisher Scientific, Fair Lawn, NJ, USA).

### Synthesis of F675-APTES/PEG-coated Mn_0.5_Zn_0.5_Fe_2_O_4_ SPIONs

Oleic acid coating was performed as described by Pawar *et al.* 66. First, 100 mg of synthesized MnZn-SPION-7s were dispersed in toluene (5 mL). Next, the MnZn-SPION-7 dispersion was mechanically stirred at room temperature for 90 min. Afterward, a mixture of ammonium hydroxide (NH_4_Cl) (750 μL) and oleic acid (OA) (3 mL) was added to the solution. The MnZn-SPION-7s were then washed thrice using a combination of acetone and a magnet. Subsequently, the MnZn-SPION-7s were dried in an oven for several minutes. Finally, a black powder consisting of OA-coated MnZn-SPION-7 was dispersed in cyclohexane for the fluorescent silica coating process. The concentration of the solution containing the OA-coated MnZn-SPION-7 was 5 mg/mL. The silica coating of MnZn-SPION-7 followed the method previously reported by Piao *et al.* 67. In this process, Flamma 647 NHS ester (10 μmol) and (3-aminopropyl) triethoxysilane (APTES) (200 μmol) were dissolved in 1 mL methanol. The solution was refrigerated and magnetically stirred for 30 h to facilitate the binding. Once all the precursors were appropriately formed, IGEPAL co-520 (2.3 g) was added to 45 mL of cyclohexane in a 100 mL round-bottom flask and magnetically stirred for 5 min at room temperature. Following this, 500 μL of OA-coated MnZn-SPION-7 solution was added to the 100 mL round-bottom flask and stirred magnetically for 5 min at room temperature, while the solution gradually turned dark brown. Ammonium hydroxide (600 µL) and tetraethyl orthosilicate (150 µL) were then incorporated into the solution, which was magnetically stirred at room temperature. After 10 h, Flamma 647-APTES solution (100 µL) and polyethylene glycol (400 µL) were added to the solution and stirred at room temperature with precaution taken to avoid lights. After an additional 2 h of stirring, the solution was washed three times by centrifugation at 7500 rpm for 5 min using acetone and ethyl ether. The resulting solution was then dispersed in distilled water.

### Confocal microscopy

GFP (U87MG cells) and tdTomato (F675-APTES/PEG-MnZn-SPION-7) were detected using an LSM510 META confocal microscope (Carl Zeiss Inc., Oberkochen, Germany) at 40× magnification. A diode and helium-neon laser were used to excite the fluorescent markers mentioned above. Cells were fixed for 10 min with 3.7% paraformaldehyde (USB, Cleveland, OH, USA). After fixation, the cells were washed with 1X PBS to remove any residual fixative. The prepared samples were mounted using the antifading reagent ProLong Gold (Invitrogen). Slides that were subjected to immunofluorescence were imaged using the same instrument.

### *In vitro* imaging system (IVIS): bioluminescence imaging

Before acquiring images, 3 mg of D-luciferin (Caliper Life Sciences, Hopkinton, MA, USA) was intraperitoneally administrated to mice (100 µL per mouse, 30 mg/ml with saline stock). Bioluminescence signals were collected after 10-30 min using the IVIS100 equipment. Gray-scale photographs of the mice were also taken, and the corresponding pseudo-color images were generated. These images were superimposed using image analysis software, including Living Image (version 2.12; Xenogen, Alameda, CA, USA) and IGOR (version 1.24; WaveMetrics, Portland, OR, USA). ROIs were defined, and the signals emitted by these ROIs were measured and expressed as photon flux in photons per second per square centimeter per steradian (photons/sec/cm^2^/sr). These measurements quantify the number of photons emitted from a unit solid angle of a sphere. To ensure accuracy, the background signal intensity was electronically subtracted from the images and measurements of the photon flux for normalization.

### *In vivo* intravital confocal microscopy

GFP-expressing U87 GBM tumors and F675-APTES/PEG-MnZn-SPION-7 in the GBM infiltration zone were imaged using intravital confocal and two-photon microscopy (IVM-CMS, IVIM Technology Inc., Daejeon, Korea). To perform repetitive time-lapse imaging of the mouse subcortex *in vivo*, a cranial imaging window was implanted as shown in Figure 6B, following a previously described method 68, 69. Briefly, the cranial bone of the anesthetized mice was exposed by skin incision, and the connective tissue covering the cranial bone was removed. Subcortical brain tissue was exposed by creating a circular hole in the cranial bone (typically 3 mm in diameter) using a dental drill (Strong 207A, Saeshin, Daegu, Korea) under a stereoscopic microscope. A 3 mm-diameter cover glass (64-0726, Warner Instruments, Holliston, MA, USA) was attached to the skull to cover the exposed subcortical brain tissue using a fast-curing adhesive (Loctite 401. Henkel, Rocky Hill, CT, USA). For protection, all exposed areas except the cover glass were covered with dental acrylic resin (1234; Lang Dental, Wheeling, IL, USA). A high-numerical-aperture (NA) water-immersion objective lens (CFI75 Apochromat 25XC W, NA1.1, Nikon, Tokyo, Japan) was used. All mice were anesthetized by intramuscular injections of zoletil (30 mg/kg) and xylazine (10 mg/kg) prior to intravital imaging. To visualize the vessels, anti-CD31 antibody (Cat no. 553708; BD Biosciences, San Jose, CA, USA) conjugated with Alexa Fluor 555 (Cat no. A20109, Thermo Fisher Scientific) was injected intravenously into the tail vein 1 h before imaging. Three lasers at wavelengths of 488, 561, and 640 nm were used to excite the fluorescence of the U87-GFP tumor, CD31-Alexa Flour 555 labeled vessels, and SPIONs, respectively.

### Statistical Analysis

All data are expressed as means ± standard deviation (SD). Statistical significance was determined using the t-test, with p < 0.05 indicating statistical significance.

## Supplementary Material

Supplementary figures and tables.

## Abbreviations

MNZN-SPION-7: 7-nm Mn_0.5_Zn_0.5_Fe_2_O_4_ SPION
AC: alternating current
AMFs: alternating magnetic fields
APCs: antigen presenting cells
B-H Curve (B-H loop): magnetic hysteresis
Magnetic Hysteresis Loop): curve
CCK-8: cell counting Kit-8
CRT: calreticulin
DAMP: danger-associated molecular pattern
DC: direct current
DCs: dendritic cells
dH: hydrodynamic diameter
DLS: dynamic light scattering
DMEM: Dulbecco's modified Eagle's medium
EDS: energy-dispersive X-ray spectroscopy
FBS: fetal bovine serum
FC/ZFC Magnetization: field cooled/zero Field cooled magnetization
Fe_3_O_4_-SPION: Fe_3_O_4_ SPION
FSaII: C3H mouse fibrosarcoma
FT: Fourier-transformed
FT-IR: Fourier-transformed infra-red
FT-RDF: Fourier-transformed radial distribution function
HMGB1: high mobility group Box 1 protein I
HSP: heat shock proteins
HTTD: high temperature thermal decomposition
IFN: interferon
IONPs: iron oxide nanoparticles
ILP: intrinsic loss power
MFs: magnetic fields
MHC (MHL): magnetic hysteresis curve (magnetic hysteresis loop)
MHT: magnetic hyperthermia
MNPs: magnetic nanoparticles
NK cells: natural killer cells
NPs: nanoparticles
NSC: neuronal stem cells
Oe: Oersted
PDI: polydispersity index
PEG: polyethylene glycol
PEG-Fe_3_O_4_-SPION: PEGylated Fe_3_O_4_ SPION
PEG-MnZn-SPION-7: PEGylated 7-nm Mn_0.5_Zn_0.5_Fe_2_O_4_ SPION
pegylated: polyethylene glycol-coated
PPMS: physical property measurement system
SAR: specific absorption ratio
SEM: scanning electron microscopy
SLP: specific loss power
SPION: superparamagnetic iron oxide nanoparticle
T: tesla
TEM: transmission electron microscopy
UVS: ultraviolet-visible spectroscopy
VSM: vibrating sample magnetometer
XAFS: X-ray Absorption Fine Structure
XANES: X-ray absorption near edge structure
XAS: X-ray absorption spectroscopy
XRD: X-ray diffraction
Z-potential: zeta-potential
