# Supplementary material for: 7-nm Mn0.5 Zn0.5Fe2O4 superparamagnetic iron oxide nanoparticle (SPION): a high-performance theranostic for MRI and hyperthermia applications
Source: Theranostics. 2025 Feb 10;15(7):2883–902. doi: 10.7150/thno.103503 (PMC11898280; doi:10.7150/thno.103503)
Supplement: Supplementary file 1 — Supplementary figures and tables. [file thnov15p2883s1.pdf]

Supplementary figures and tables with legends

SUPPLEMENTARY TABLES

	Fe^{3+}	Mn^{2+}	Zn^{2+}	1,2-HDD	Oleic Acid	Oleylamine	Benzyl Ether
X=0	2 mmol	0 mmol	1.0 mmol	10 mmol	6 mmol	6 mmol	20 mL
X=0.1	2 mmol	0.1 mmol	0.9 mmol	10 mmol	6 mmol	6 mmol	20 mL
X=0.2	2 mmol	0.2 mmol	0.8 mmol	10 mmol	6 mmol	6 mmol	20 mL
X=0.3	2 mmol	0.3 mmol	0.7 mmol	10 mmol	6 mmol	6 mmol	20 mL
X=0.4	2 mmol	0.4 mmol	0.6 mmol	10 mmol	6 mmol	6 mmol	20 mL
X=0.5	2 mmol	0.5 mmol	0.5 mmol	10 mmol	6 mmol	6 mmol	20 mL
X=0.6	2 mmol	0.6 mmol	0.4 mmol	10 mmol	6 mmol	6 mmol	20 mL
X=0.7	2 mmol	0.7 mmol	0.3 mmol	10 mmol	6 mmol	6 mmol	20 mL
X=0.8	2 mmol	0.8 mmol	0.2 mmol	10 mmol	6 mmol	6 mmol	20 mL
X=0.9	2 mmol	0.9 mmol	0.1 mmol	10 mmol	6 mmol	6 mmol	20 mL
X=1	2 mmol	1.0 mmol	0 mmol	10 mmol	6 mmol	6 mmol	20 mL

Supplementary Table 1. Composition of reagents for the synthesis of manganese- and zinc-doped iron oxide nanoparticles ($Mn_xZn_{1-x}Fe_2O_4$, where x ranges from 0 to 1). 1,2-HDD: 1.2 hexadecanediol.

Sample	Mean Diameter	Standard Deviation	Particles Counted
X=0	7.905 nm	1.939	100
X=0.1	8.144 nm	1.965	100
X=0.2	8.208 nm	1.706	100
X=0.3	8.930 nm	1.649	100
X=0.4	9.081 nm	1.044	100
X=0.5	7.008 nm	1.434	100
X=0.6	7.739 nm	1.412	100
X=0.7	8.818 nm	2.027	100
X=0.8	7.867 nm	1.609	100
X=0.9	7.641 nm	1.980	100
X=1	8.811 nm	1.561	100

Supplementary Table 2. Mean diameters (numerical value) of the synthesized $Mn_xZn_{1-x}Fe_2O_4$

nanoparticles (where x ranges from 0 to 1) determined by energy-dispersive X-ray spectroscopy (EDS).

SUPPLEMENTARY FIGURES

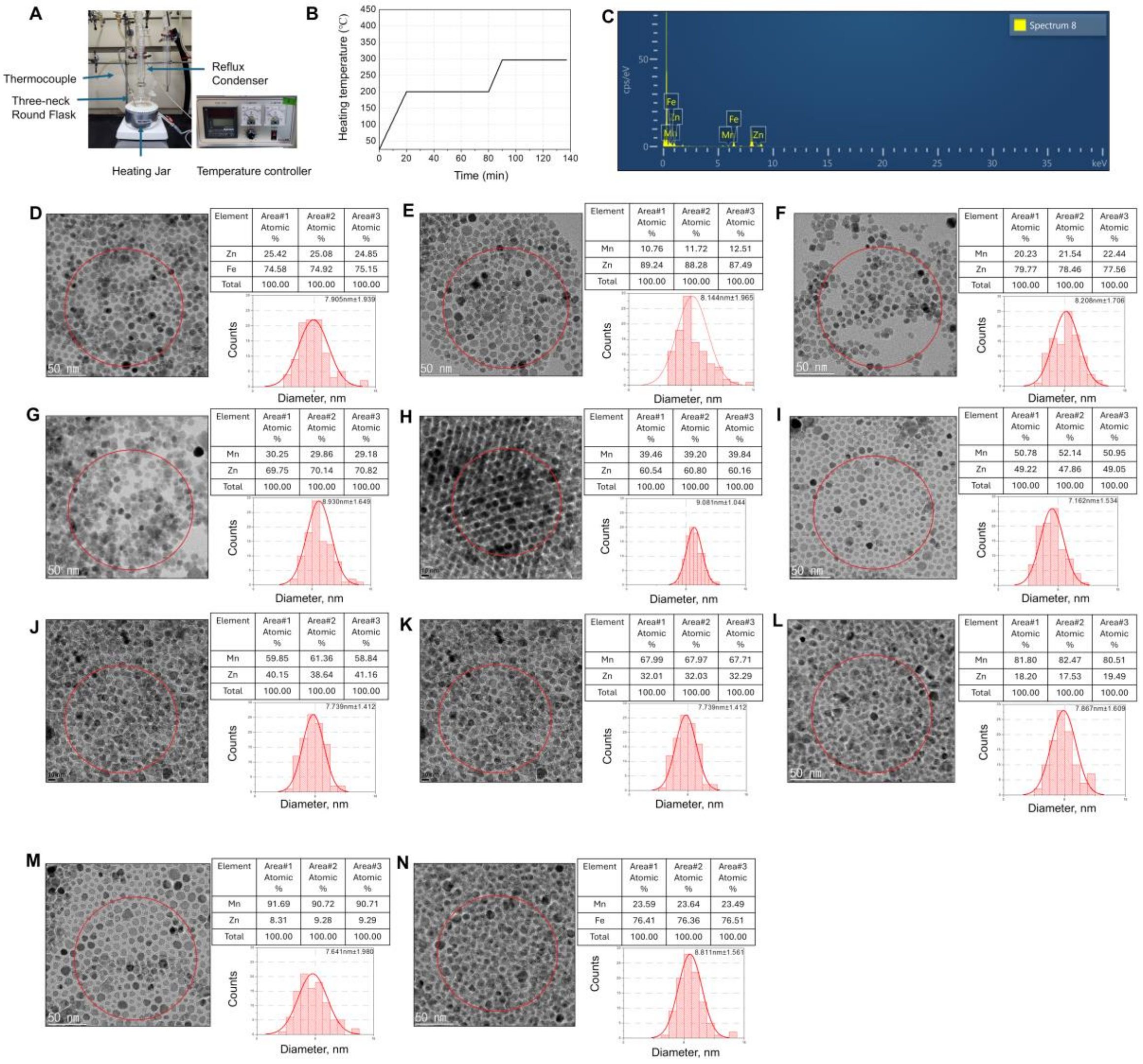

Figure S1. Transmission electron microscopy (TEM) and energy dispersive X-ray spectroscopy (EDS) results of synthesized $Mn_xZn_{1-x}Fe_2O_4$ ($_{X=0\sim1,\ 1-X=1\sim0}$) nanoparticles. **A:** The setup for the synthesis of $Mn_xZn_{1-x}Fe_2O_4$ nanoparticles using the modified high-temperature thermal decomposition (mHTTD) method includes a thermocouple, a reflux condenser, a three-neck round flask, a heating jar, a magnetic stirring bar (located inside the flask), and a temperature controller. **B:** Schematic diagram of temperature changes over time illustrating mHTTD method for the synthesis of $Mn_xZn_{1-x}Fe_2O_4$ nanoparticles. **C:** Representative spectrum

data determined by EDS to determine Zn and Mn ratio. **D–N:** The image and mean diameter (numerical value) of the synthesized nanoparticle variants obtained using TEM, listed as follows: **D:** $ZnFe_2O_4$ (7.905 ± 1.939 nm), **E:** $Mn_{0.1}Zn_{0.9}Fe_2O_4$ (8.144 ± 1.965 nm), **F:** $Mn_{0.2}Zn_{0.8}Fe_2O_4$ (8.208 ± 1.706 nm), **G:** $Mn_{0.3}Zn_{0.7}Fe_2O_4$ (8.930 ± 1.649 nm), **H:** $Mn_{0.4}Zn_{0.6}Fe_2O_4$ (9.081 ± 1.044 nm), **I:** $Mn_{0.5}Zn_{0.5}Fe_2O_4$ (7.08 ± 1.434 nm) **J:** $Mn_{0.6}Zn_{0.4}Fe_2O_4$ (7.739 ± 1.412 nm) **K:** $Mn_{0.7}Zn_{0.3}Fe_2O_4$ (8.818 ± 2.027 nm), **L:** $Mn_{0.8}Zn_{0.2}Fe_2O_4$ (7.867 ± 1.609 nm), **M:** $Mn_{0.9}Zn_{0.1}Fe_2O_4$ (7.641 ± 1.980 nm), **N:** $MnFe_2O_4$ (8.811 ± 1.561 nm).

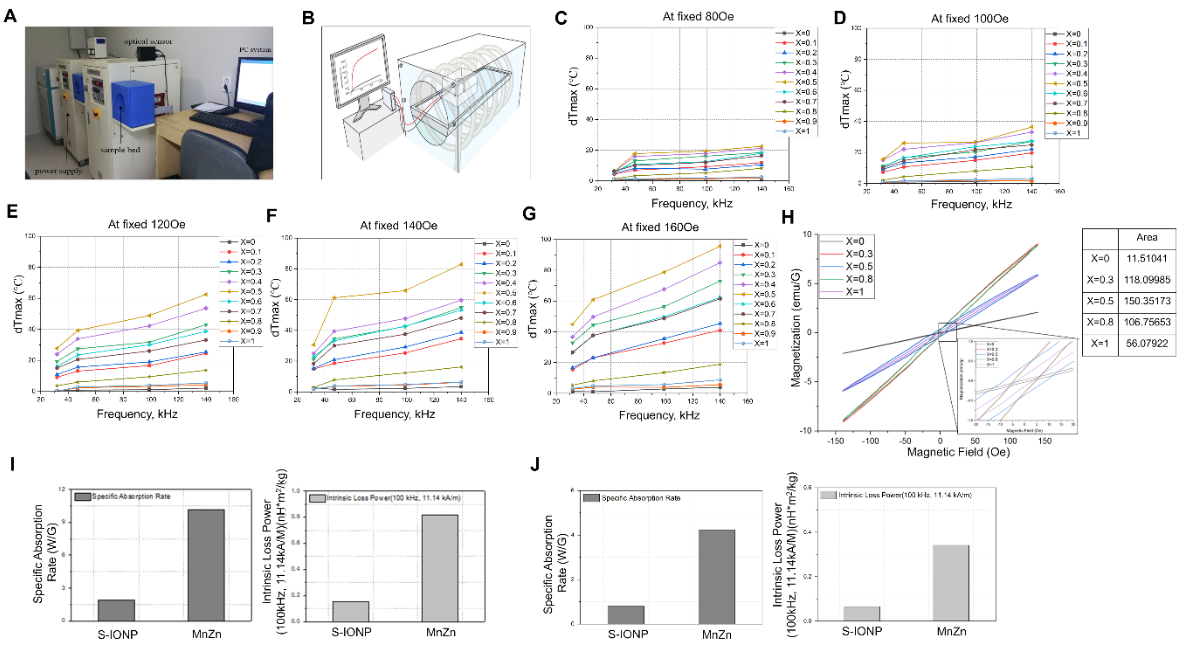

Figure S2. Physical properties of $Mn_xZn_{1-x}Fe_2O_4$ (x=0~1, 1-x=1~0) nanoparticles. A: An alternating magnetic field (AMF) generation system equipped with an optical sensor, a sample bed, a power supply, and an operating PC. **B:** Schematic illustration of the measurement of hyperthermia induced by magnetic nanoparticles exposed to an AMF. **C–G:** Heating characteristics of synthesized $Mn_xZn_{1-x}Fe_2O_4$ nanoparticles (where x ranges from 0 to 1). Eleven $Mn_xZn_{1-x}Fe_2O_4$ nanoparticles were studied under eight different frequencies (30, 50, 100, 140, 170, 200, 240, and 360 KHz) with five different magnetic field strengths: **C:** 80 Oe, **D:** 100 Oe, **E:**120 Oe, **F:** 140 Oe, and **G:** 160 Oe. All were measured with a duration of 600 s. **H:** The area of the synthesized solid-state $Mn_xZn_{1-x}Fe_2O_4$ nanoparticles exhibited the highest value of 150.35173 when compared to other $Mn_xZn_{1-x}Fe_2O_4$ nanoparticles in the minor loop hysteresis under an AMF at a fixed AC frequency of 100 kHz and a magnetic field strength of 140 Oe. **I, J:** Comparison of specific absorption rates (SAR) (W/g) and intrinsic loss of power

(ILP) (nHm^2/kg) of MnZn-SPION-7 and Fe_3O_4-SPION under an AMF of 140 Oe and an AC of 100 kHz as a powder (**I**), or in solution (**J**). In powdered form, the SAR of MnZn-SPION-7 and Fe_3O_4-SPION are 10 and 2 W/g, respectively and ILP 0.82 and 0.15 nHm^2/kg, respectively. In solution form, the SAR value for MnZn-SPION-7 and Fe_3O_4-SPION are 4.38 and 0.97 W/g, and ILP 0.12 and 0.34 nHm^2/kg, respectively.

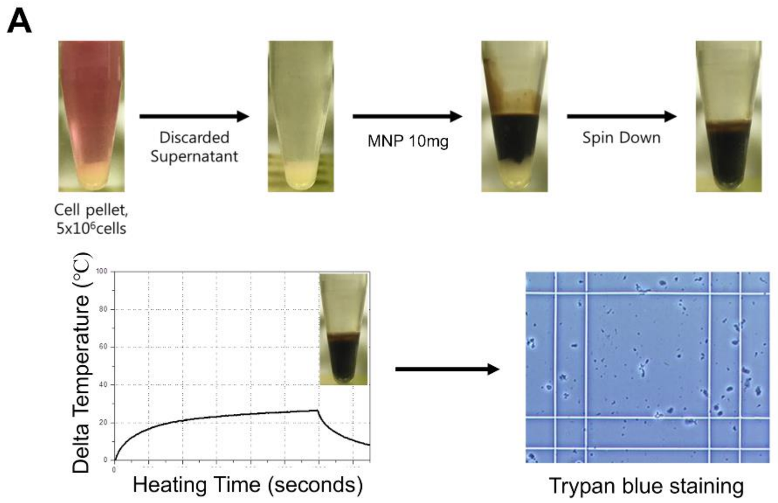

Figure S3. The temperature (26.6 °C) of 5×10^6 cells of U87MG suspended in aqueous fluids of 10 mg/mL PEG-MnZn-SPION-7 in an Eppendorf® tube exposed to an AMF at 100 kHz and 140 Oe for 20 mins and trypan blue staining, revealing that all the cells were killed. T2 signal measurements of PEG-MnZn-SPION-7 using a 3 T MRI system in comparison with PEG-Fe_3O_4-SPION-7.

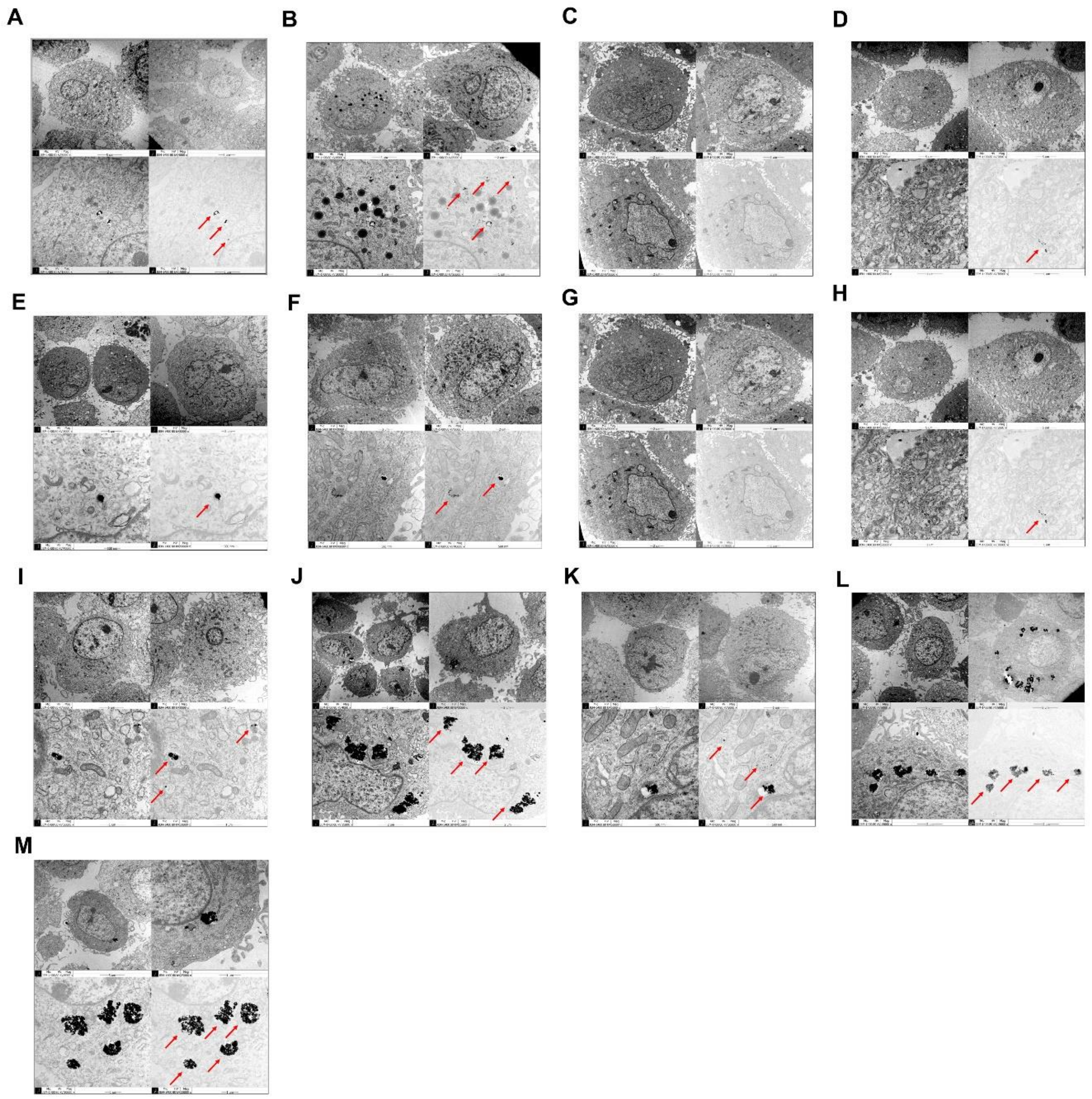

Figure S4. TEM images of nine glioblastoma cell lines and two primary cultured human normal cortex cells treated with 100 μg/mL PEG-MnZn-SPION-7 at a magnification of 500,000×. **A.** U87MG with PEG-MnZn-SPION-7. **B:** U373 with PEG-MnZn-SPION-7. **C:** NSC-09 with PEG-MnZn-SPION-7. **D:** NSC-10 with PEG-MnZn-SPION-7. **E:** GBL-28 with PEG-MnZn-SPION-7. **F:** GBL-37 with PEG-MnZn-SPION-7. **G:** NSC-09 with PEG-MnZn-SPION-7. **H:** NSC-10 with PEG-MnZn-SPION-7. **I:** U118 with PEG-MnZn-SPION-7. **J:** U138 with PEG-MnZn-SPION-7. **K:** A172 with PEG-MnZn-SPION-7. **L:** U251 with PEG-MnZn-SPION-7. **M:** T98G with PEG-MnZn-SPION-7

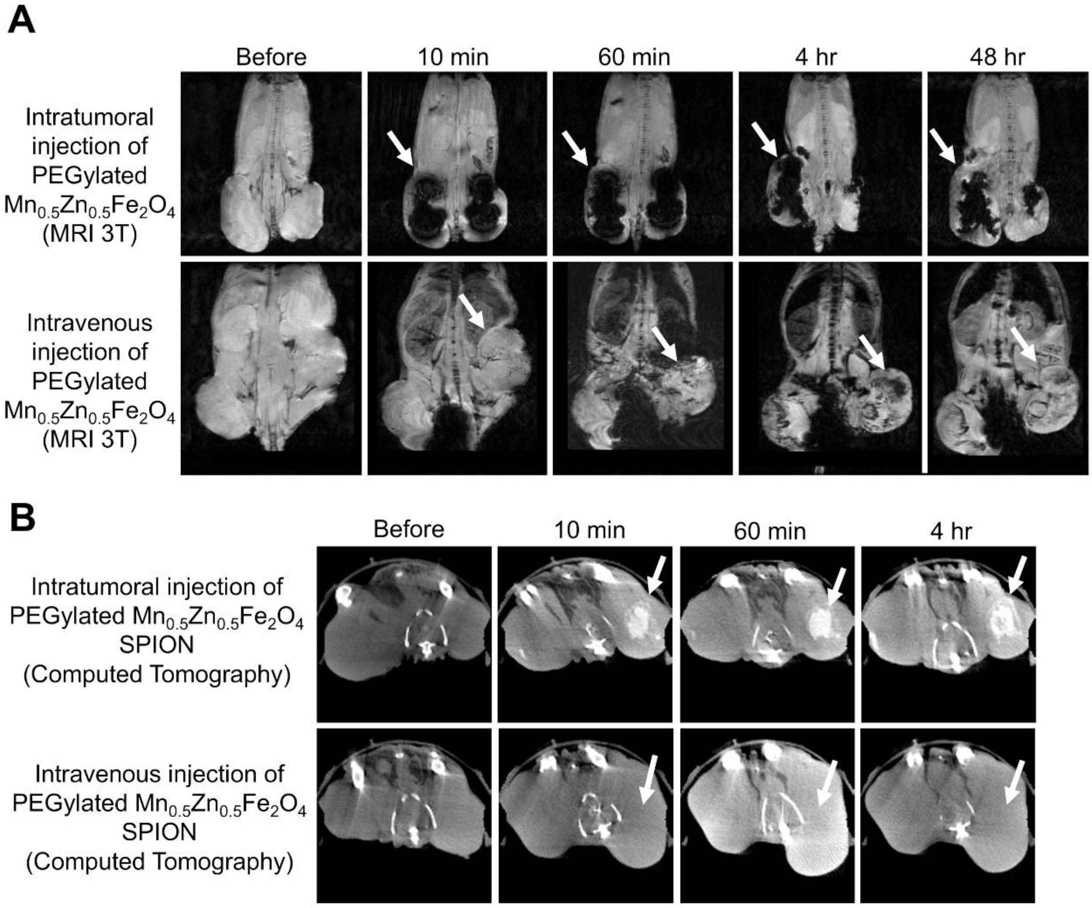

Figure S5. 3 T magnetic resonance images (MRI) and computed tomography (CT) obtained before and after intratumoral or intravenous injection of PEG-MnZn-SPION-7 in the nude mouse subcutaneous fibrosarcoma (FSaII) model at various times points (10 min, 1 h, 4 h, and 2 d). **A:** 3 T brain MRI showing significant contrast enhancement (indicated by arrows) in the tumors following both intratumoral or intravenous injection of PEG-MnZn-SPION-7 in the FSaII model in nude mice. **B:** CT images demonstrating significant contrast enhancement in tumors following intratumoral or intravenous injection of PEG-MnZn-SPION-7 in the subcutaneous fibrosarcoma (FSaII*) model in nude mice. (* FSaII tumor is a radiation-induced fibrosarcoma of C3H mice, originally obtained from the Dr. H. Suit's laboratory at the Massachusetts General Hospital. Early-generation FSaII cells were provided by Dr. C.W. Song of the University of Minnesota).

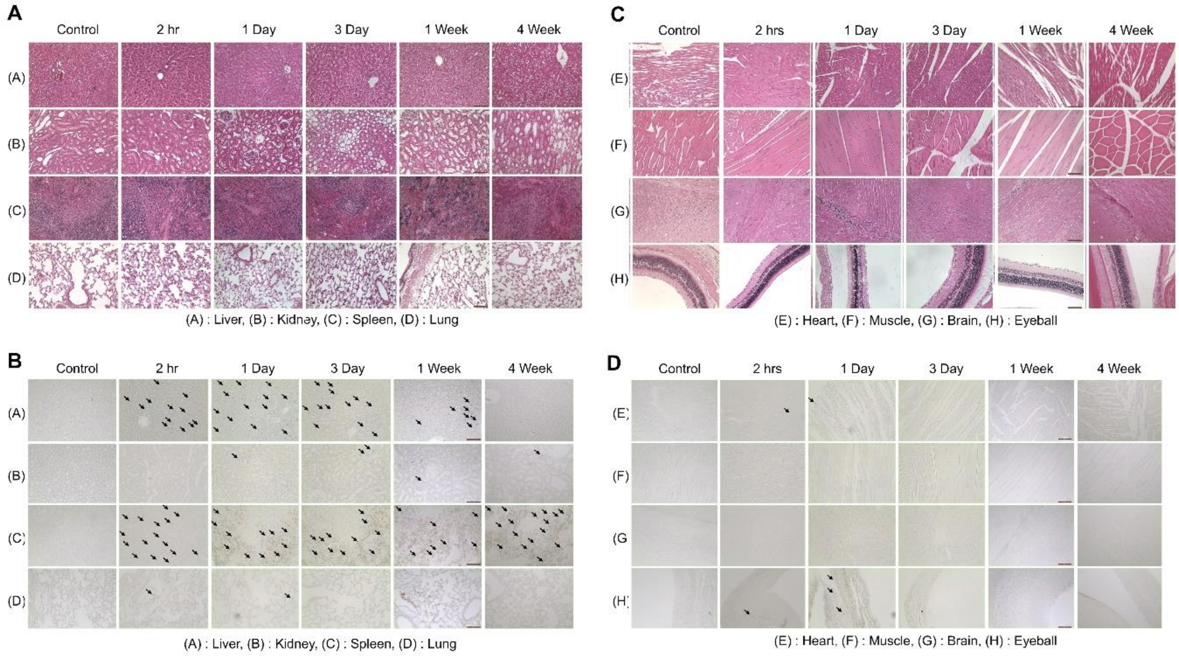

Figure S6. H&E and Prussian blue staining performed on tissues from the liver, kidney, spleen, lung, heart, muscle, brain, and eyeball after intravenous injection of PEG-MnZn-SPION-7 in nude mice. **A, B:** H&E staining of tissues from the liver, kidney, spleen, lung, heart, muscle, brain, and eyeball were conducted before injection and at 2 h, 1 d, and 3 d, 1 week, and 4 weeks after intravenous injection of PEG-MnZn-SPION-7 in nude mice. No tissue damage was observed following intravenous injection of PEG-MnZn-SPION-7 in nude mice. **C, D**: Prussian blue staining of tissues from the liver, kidney, spleen, lung, heart, muscle, brain, and eyeball was carried out before injection and at two hours, one day, and 3 d, 1w, and 4 w after intravenous injection of PEG-MnZn-SPION-7 in nude mice. Most of the PEG-MnZn-SPION-7 were cleared from the tissues, except for the spleen and kidney, in four weeks after intravenous injection. Black arrows in **B** and **D** indicate PEG-MnZn-SPION-7 within each tissue. *n = 4*

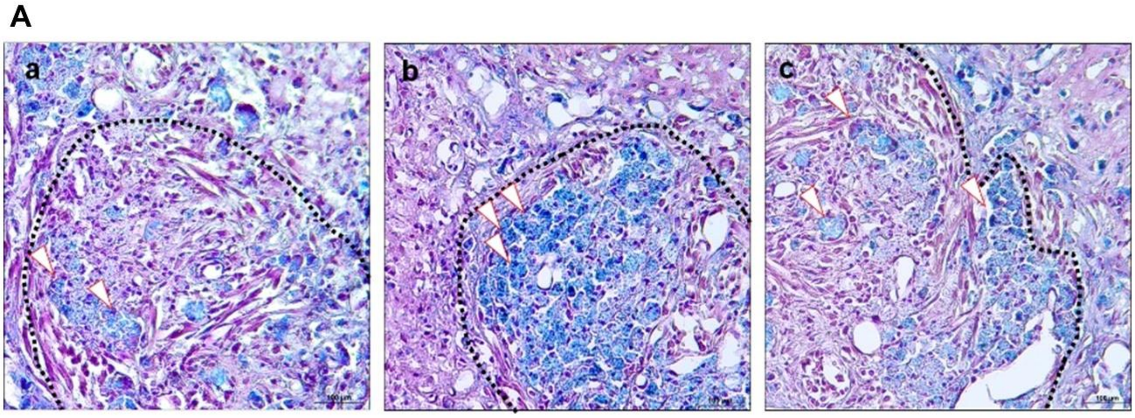

Figure S7. Representative images of Prussian blue staining from individual mice **A–C:** showing MNP-injected tumors in the cortex of nude mice, two days after injection with 5 μL PEG-MnZn-SPION-7 (30 mg/mL) *in vivo* nude mouse orthotopic brain tumor models of U87MG. Red arrows highlight iron-containing cancer cells, while black dotted lines outline the approximate tumor stromal regions defined by fibroblast cell linings for improved visualization. *n* = *4*.

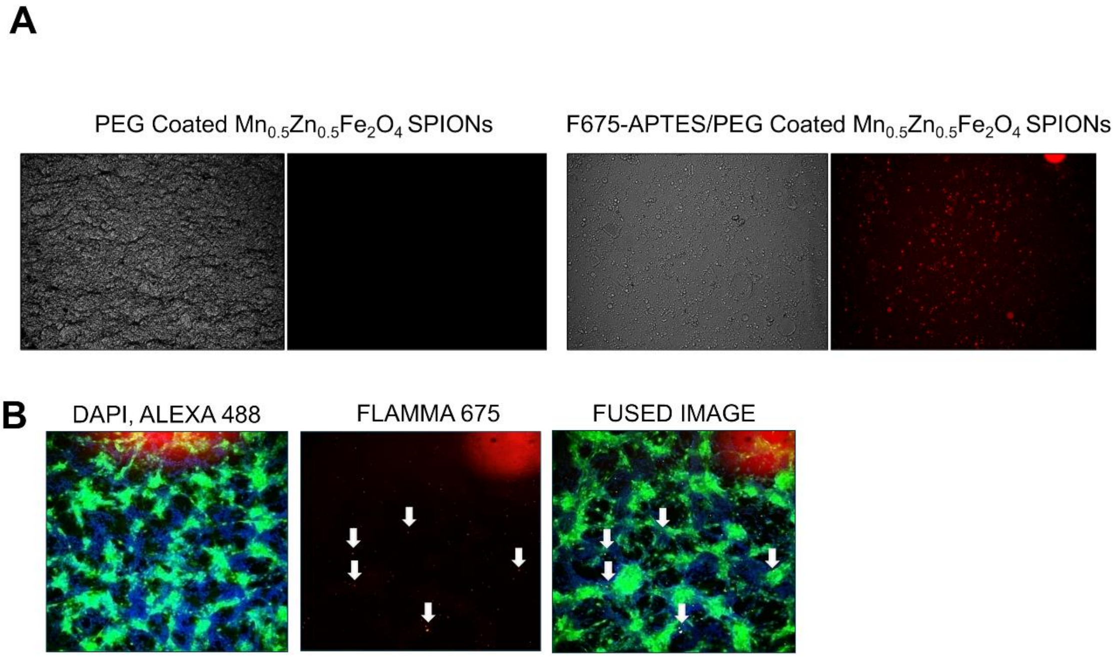

Figure S8. PEG-MnZn-SPION-7 coated with F675-APTES showing red fluorescence. **A:**

SEM images of PEG-MnZn-SPION-7 (left) and F675-APTES/PEG-MnZn-SPION-7 (right) to confirm fluorescence. **B:** Intravital confocal microscope image of F675-APTES/PEG-MnZn-SPION-7 along with GFP expressing U87 tumor cell (Alexa 488) and DAPI.

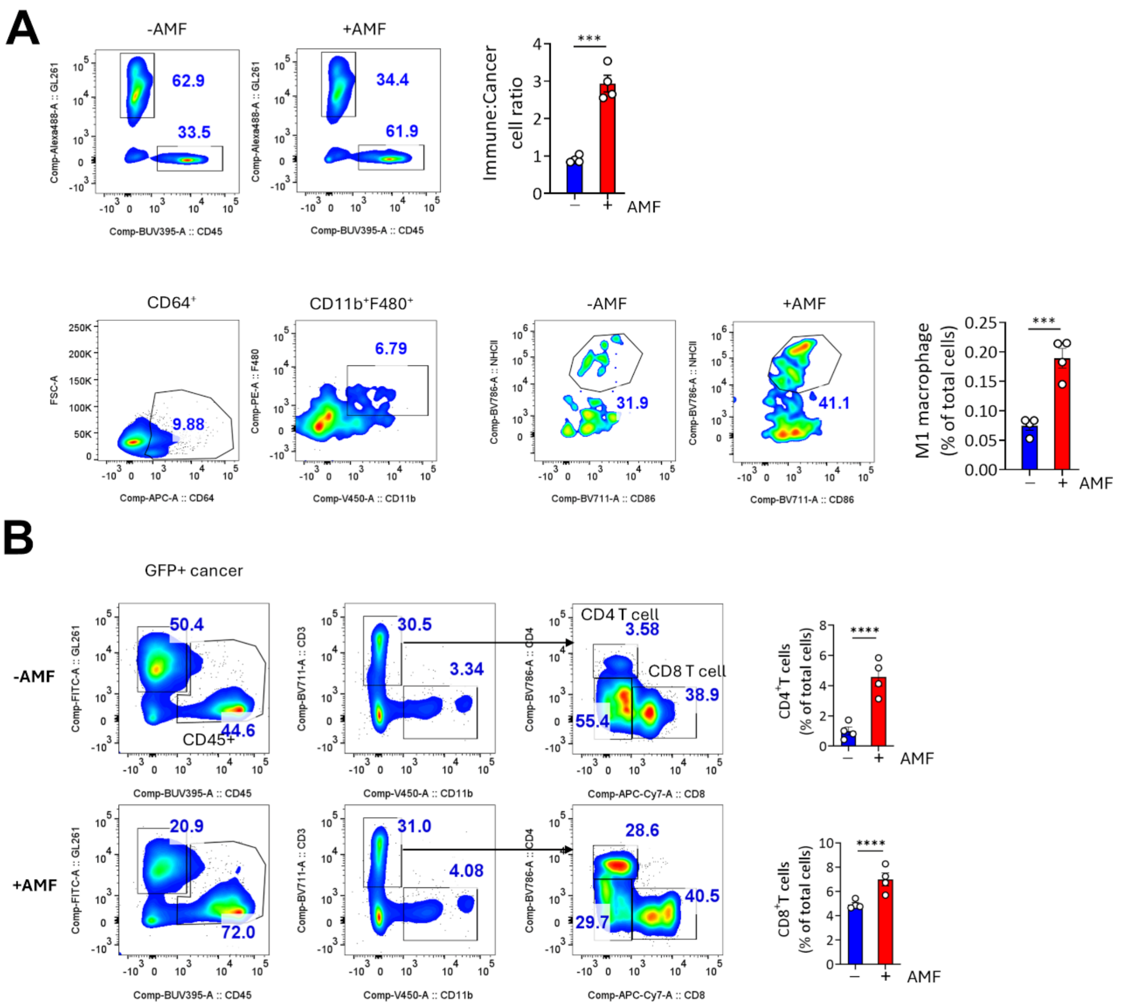

Figure S9. Flow cytometry analysis of splenocyte co-culture with GL-261 cells (2 mg/mL) following *in vitro* MHT treatment. **A:** Surface staining showing the immune-to-cancer cell ratio (top) and **B:** the gating strategy for identifying $CD86^{+}MHCII^{hi}$ M1 macrophages in AMF-treated and non-treated groups. **C:** Gating strategy to identify $CD3^{+}$ T cells and their subsets, $CD4^{+}$ and $CD8^{+}$ T cells. The bar graph on the right shows the population differences between the two groups for each T cell subset. *** $p < 0.005$, **** $p < 0.001$ (multi
